# Perioperative PD-1/PD-L1 inhibitors for resectable non-small cell lung cancer: A meta-analysis based on randomized controlled trials

**DOI:** 10.1371/journal.pone.0310808

**Published:** 2024-09-23

**Authors:** Hai Huang, Lianyun Li, Ling Tong, Houfu Luo, Huijing Luo, Qimin Zhang

**Affiliations:** Department of Oncology, Taihe People’s Hospital, Taihe, China; Abu Dhabi University, UNITED ARAB EMIRATES

## Abstract

**Background:**

PD-1/PD-L1 inhibitors (PI) have shown promising results in both neoadjuvant and adjuvant therapies for resectable non-small cell lung cancer (NSCLC). However, substantial evidence from large-scale studies is still lacking for their use in the perioperative setting (neoadjuvant plus adjuvant). This meta-analysis aims to evaluate the integration of perioperative PI (PPI) with neoadjuvant chemotherapy for resectable NSCLC.

**Methods:**

To identify appropriate randomized controlled trials (RCTs), we thoroughly explored six different databases. The primary endpoint was survival, while the secondary measures included pathological responses and adverse events (AEs).

**Results:**

Six RCTs involving 2941 patients were included. The PPI group significantly improved overall survival (OS) (hazard ratio [HR]: 0.62 [0.51, 0.77]), event-free survival (EFS) (HR: 0.57 [0.51, 0.64]), pathological complete response (risk ratio [RR]: 5.81 [4.47, 7.57]), and major pathological response (RR: 2.60 [1.77, 3.82]). Benefits in EFS were seen across all subgroups. OS rates at 12–48 months and EFS rates at 6–48 months were higher in the PPI cohort. Furthermore, the advantages in OS and EFS increased with prolonged survival times. The PPI group also exhibited higher rates of surgery and R0 resections. However, the PPI group experienced more grade 3–5 AEs, serious AEs, and treatment discontinuations due to AEs.

**Conclusions:**

The integration of perioperative PI with neoadjuvant chemotherapy can significantly improve survival and pathological responses for resectable NSCLC. However, the increased incidence of grade 3–5 AEs must be carefully evaluated.

## Introduction

Lung cancer remains a major cause of cancer-related deaths globally, with non-small cell lung cancer (NSCLC) accounting for approximately 85% of all cases [1]. Among NSCLC patients, those with resectable disease present a unique opportunity for curative surgical intervention. However, the high recurrence rates following surgery underscore the need for effective perioperative treatment strategies [2]. Recently, PD-1/PD-L1 inhibitors (PI) have emerged as promising therapeutic options, transforming the treatment landscape for NSCLC [3]. Various studies have confirmed the efficacy of PI in both neoadjuvant and adjuvant settings [4, 5]. For instance, neoadjuvant therapy with PI has demonstrated a reduction in tumor burden and improved surgical outcomes by enhancing pathological responses [4]. Similarly, adjuvant therapy with these inhibitors has shown improved survival rates by targeting residual disease and preventing recurrence [5]. Pasqualotto et al.’s meta-analysis, based on seven RCTs, also confirmed the role of PI in both standalone neoadjuvant and adjuvant therapy for resectable NSCLC [6]. Despite these successes, significant controversies and gaps in evidence remain, particularly regarding the integration of these treatments into a comprehensive perioperative approach.

One of the primary controversies in this field involves the optimal timing and sequencing of PI in conjunction with chemotherapy [7]. While neoadjuvant chemotherapy has long been a standard to downstage tumors and eradicate micrometastases, the addition of PI in both the neoadjuvant and adjuvant settings (perioperative PI [PPI]) has not been thoroughly investigated in large sample meta-analyses. The hypothesis that combining chemotherapy with PI can elicit a stronger anti-tumor immune response is compelling [8–13]. Chemotherapy can cause immunogenic cell death, which potentially enhances the efficacy of PI by increasing tumor antigen presentation and T-cell infiltration. However, this theoretical synergy requires validation through rigorous clinical trials [14].

By pooling data from high-quality randomized controlled trials (RCTs), our analysis aims to provide a comprehensive assessment of the impact of PPI on key outcomes, including overall survival (OS), event-free survival (EFS), pathological response, and adverse events (AEs).

## Materials and methods

### Search strategy

MeSH terms such as “PD-1/PD-L1 (see S1 Table for details)”, “Chemotherapy”, “Lung cancer”, and “Randomized” were utilized. We thoroughly searched six databases, including PubMed, ScienceDirect, the Cochrane Library, Scopus, EMBASE, and Web of Science. The search period covered from inception to June 15, 2024 (**S1 Table**). Furthermore, the reference lists of the selected studies were scrutinized to identify additional eligible RCTs.

### Selection criteria

Inclusion criteria (PICOS):

1. Participants (P): resectable NSCLC.
2. Intervention (I) and control (C): PPI group (PPI plus neoadjuvant chemotherapy) versus Chemotherapy group (neoadjuvant chemotherapy).
3. Outcomes (O): survival, surgery condition, pathological response, and safety.
4. Study design (S): RCTs.

Exclusion criteria: animal experiments, reviews, meta-analyses, case reports, and studies missing key data.

### Data extraction

Data included study characteristics (phase, period, etc.), patient demographics (sex, histologic classification, etc.), survival metrics (OS and EFS), survival rates (OS rate [OSR] and EFS rate [EFSR]), pathological responses (objective response rate [ORR], major pathologic response [MPR], etc.), and AEs (total, grade 3–5, etc.). Data were independently extracted by two researchers, and discrepancies were resolved through re-evaluation (**S1 File**).

### Outcome assessments

The OSR and EFSR were analyzed at 6–48 months. Subgroup analyses of EFS were conducted according to age, sex, smoking status, Eastern Cooperative Oncology Group Performance Status (ECOG PS), race, geographic region, pathological stage, histologic classification, and PD-L1 TPS.

### Quality assessment

We employed the Cochrane Risk Assessment Tool and the Jadad scale to evaluate the quality of RCTs, with the latter rating studies up to 5 points based on randomization, blinding, and participant inclusion, considering scores of 3 or more as high quality [15, 16]. The GRADE approach was used to assess the reliability of the results [17].

### Statistical analysis

Review Manager 5.3, Stata 12.0 and SPSS 15.0 were used for data analysis. We employed hazard ratios (HR) for evaluating survival outcomes and risk ratios (RR) for dichotomous outcomes. Heterogeneity was assessed using the *I*^2^ statistic and the *χ*^2^ test. A fixed-effects model was selected when *I*^2^ was less than 50% or the P-value was above 0.1, indicating low heterogeneity; otherwise, a random-effects model was used. Funnel plots were used to assess publication bias. Statistical significance was defined as P < 0.05. (PROSPERO ID: CRD42024563648).

## Results

### Search results

**Fig 1:**
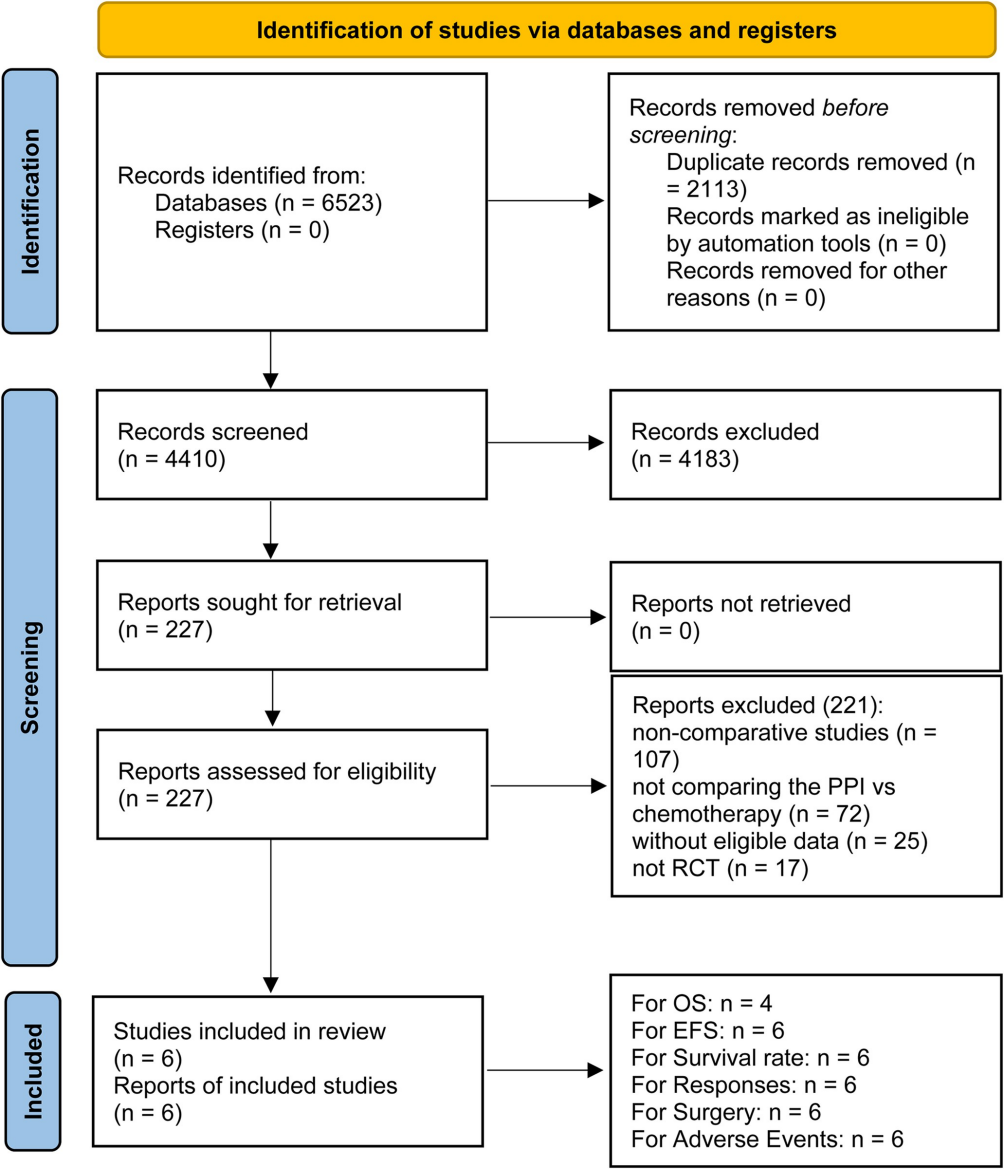
Flow chart.

Our meta-analysis incorporated RCTs involving 2941 patients: AEGEAN, CheckMate 77T, KEYNOTE-671, NADIM II, Neotorch, and RATIONALE-315 (**Fig 1**) [8–13]. **Table 1** presents an overview of the baseline characteristics of these studies. Among these, three were global multicenter trials [8–10], two were conducted in China [12, 13], and one was based in Spain [11]. According to **S1 Fig** and **S2 Table**, all studies exhibited high quality. The GRADE approach was utilized to assess the quality of the results, which ranged from medium to high (**S3 Table**).

**Table 1:** Baseline characteristics of the included studies.

Study		Phase	Period	Country	Groups	Patients	Sex (M/F)	Age (Mean, year)	ECOG PS		Histologic classification		TNM Stage			PD-1/PD-L1 type	Follow up (months)
									0	1	SCC	Non-SCC	II	IIIA	IIIB		
AEGEAN (NCT03800134)	Heymach 2023 [8]	III	2019.01–2022.04	Global multicenter	PPI	366	252/114	65	251	115	169	196	104	173	88	Durvalumab	34.0
					Chemotherapy	374	278/96	65	255	119	191	179	110	165	98		
CheckMate 77T (NCT04025879)	Cascone 2024 [9]	III	2019.11–2022.04	Global multicenter	PPI	229	167/62	66	147	82	116	113	81	146		Nivolumab	25.4
					Chemotherapy	232	160/72	66	141	91	118	114	81	149			
KEYNOTE-671 (NCT03425643)	Wakelee 2023 [10]	III	2018.04–2021.12	Global multicenter	PPI	397	279/118	63	253	144	226	171	118	217	62	Pembrolizumab	25.2
					Chemotherapy	400	284/116	64	246	154	173	227	121	225	54		
NADIM II (NCT03838159)	Provencio 2023 [11]	II	2019.06–2021.02	Spain	PPI	57	36/21	65	31	26	21	36	0	44	13	Nivolumab	26.1
					Chemotherapy	29	16/13	63	16	13	14	15	0	24	5		
Neotorch (NCT04158440)	Lu 2024 [12]	III	2020.03–2023.06	China	PPI	202	181/21	62	70	132	157	45	0	136	66	Toripalimab	18.3
					Chemotherapy	202	189/13	61	73	129	157	45	0	137	65		
RATIONALE-315 (NCT04379635)	Zhang 2023 [13]	III	2020.05–2023.08	China	PPI	226	205/21	62	143	83	179	45	93	133	0	Tislelizumab	22.0
					Chemotherapy	227	205/22	63	154	73	175	50	92	135	0		

**Abbreviations:** AE: Adverse event; ECOG PS: Eastern Cooperative Oncology Group Performance Status; PD-1: Programmed cell death protein 1; PD-L1: Programmed cell death 1 ligand 1; PPI: Perioperative PD-1/PD-L1 inhibitors; SCC: Squamous cell carcinoma; TNM: Tumor Node Metastasis.

### Survival

**Fig 2:**
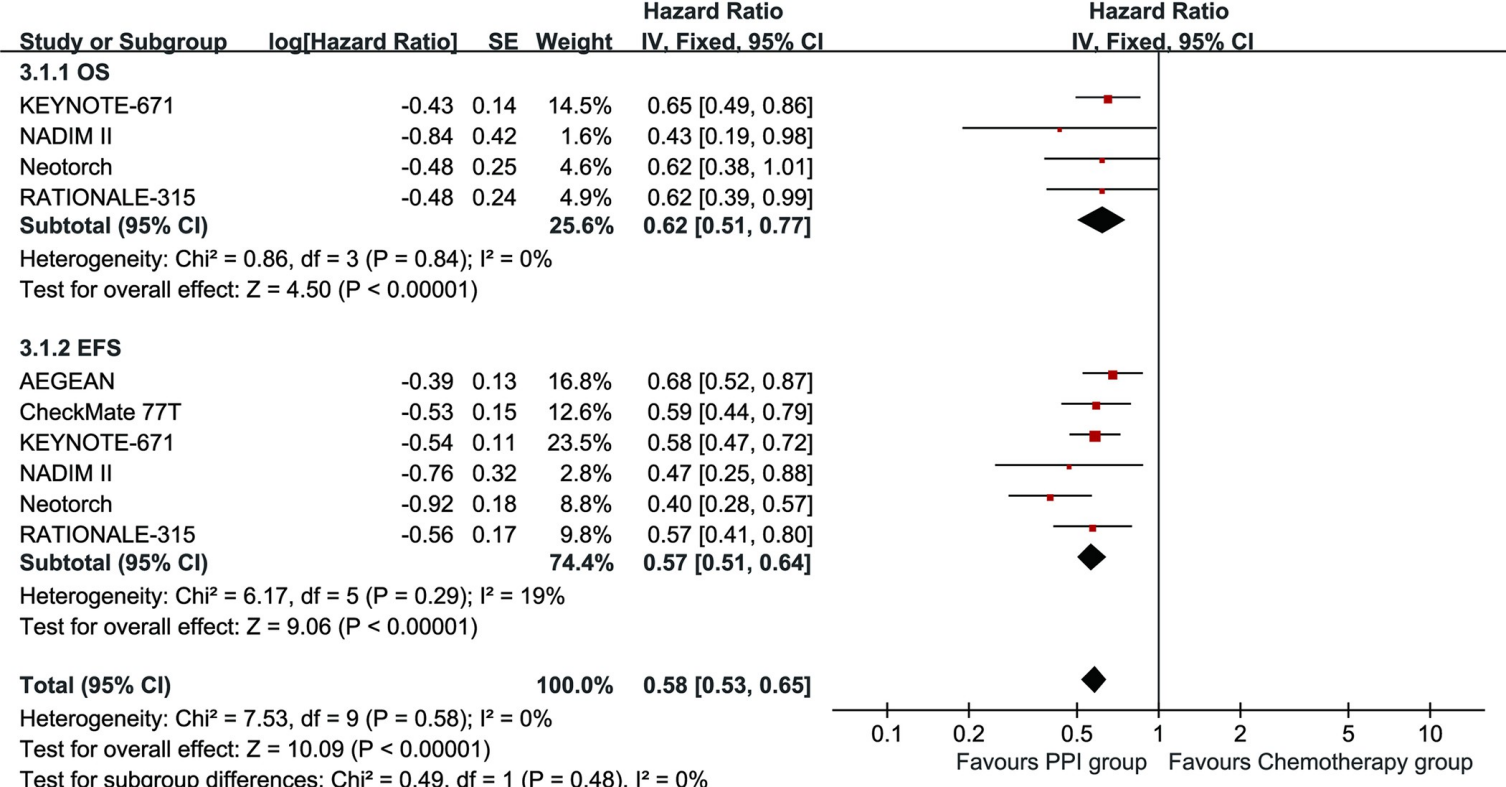
Forest plots of overall survival and event-free survival associated with PPI versus chemotherapy.

**Fig 3:**
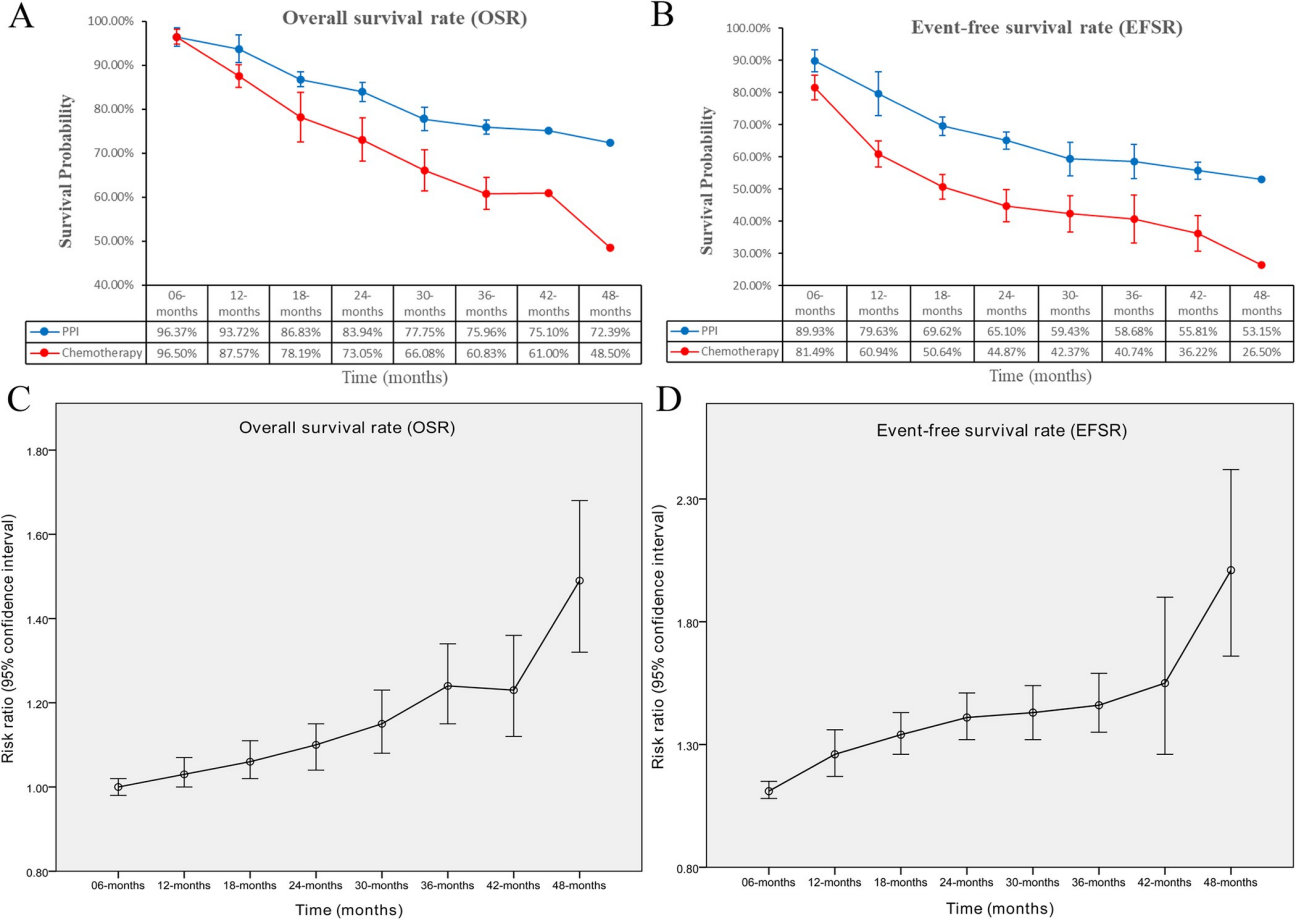
Comparisons of OSR and EFSR. (A) OSR at 6–48 months between the two groups; (B) EFSR at 6–48 months between the two groups; (C) trend of risk ratios in OSR; (D) trend of risk ratios in EFSR.

In the PPI group, OS improved significantly, with an HR of 0.62 [0.51, 0.77] (**Fig 2**). Additionally, the OSR at 12 to 48 months was higher in this group (**S2 Fig**). The benefits in OSR became more evident as the survival time lengthened (**Fig 3A and 3C**).

In the PPI group, EFS also improved significantly, with an HR of 0.57 [0.51, 0.64] (**Fig 2**). The EFSR at 6 to 48 months was higher in the PPI group (**S3 Fig**). The benefits in EFSR became more evident as the survival time lengthened (**Fig 3B and 3D**). The EFS advantage of the PPI group was consistent across all subgroups, particularly in the PD-L1 TPS > 50% subgroup (HR: 0.45 [0.35, 0.58]) (**Table 2**).

**Table 2:** Subgroup analysis of event-free survival.

Subgroups	Event-free survival				
	Included studies	Patients	HR (95% CI)	*I* ^2^	*P*
**All patients**	6	2941	0.57 [0.51, 0.65]	18%	<0.00001
**Age (year)**					
< 65	6	1631	0.55 [0.46, 0.65]	0%	<0.00001
> 65	6	1310	0.59 [0.49, 0.70]	0%	<0.00001
**Sex**					
Female	5	655	0.64 [0.49, 0.84]	26%	0.001
Male	5	2200	0.56 [0.48, 0.64]	29%	<0.00001
**Smoking status**					
Active smoker	4	535	0.52 [0.40, 0.70]	0%	<0.00001
Former smoker	5	2000	0.55 [0.44, 0.69]	54%	<0.00001
Non-smoker	6	406	0.62 [0.45, 0.87]	43%	0.006
**ECOG PS**					
0	4	1233	0.58 [0.48, 0.71]	0%	<0.00001
1	4	824	0.56 [0.44, 0.71]	48%	<0.00001
**Race category**					
White	2	536	0.53 [0.41, 0.68]	0%	<0.00001
Asian	4	1508	0.54 [0.45, 0.65]	27%	<0.00001
Others	3	1033	0.60 [0.49, 0.73]	35%	<0.00001
**Geographic region**					
Asia	4	1377	0.51 [0.42, 0.62]	5%	<0.00001
Europe	3	617	0.63 [0.48, 0.83]	0%	0.0008
North America	2	130	0.64 [0.34, 1.18]	0%	0.15
**Pathological stage (TNM)**					
II	4	798	0.66 [0.51, 0.86]	0%	0.002
III	6	2134	0.52 [0.45, 0.60]	0%	<0.00001
**Histologic classification**					
Nonsquamous	6	1291	0.62 [0.52, 0.74]	0%	<0.00001
Squamous	6	1641	0.53 [0.45, 0.63]	30%	<0.00001
**PD-L1 TPS**					
<1%	5	781	0.75 [0.60, 0.94]	0%	0.01
>1%	4	1071	0.47 [0.39, 0.58]	0%	<0.00001
1–49%	5	944	0.52 [0.37, 0.72]	54%	<0.00001
>50%	5	840	0.45 [0.35, 0.58]	37%	<0.00001

**Abbreviations:** CI: Confidence interval; ECOG PS: Eastern Cooperative Oncology Group Performance Status; HR: Hazard ratio; PD-1: Programmed cell death protein 1; PD-L1: Programmed cell death 1 ligand 1; PPI: Perioperative PD-1/PD-L1 inhibitors; TNM: Tumor Node Metastasis; TPS: Tumor cell proportion score.

### Pathological responses

**Fig 4:**
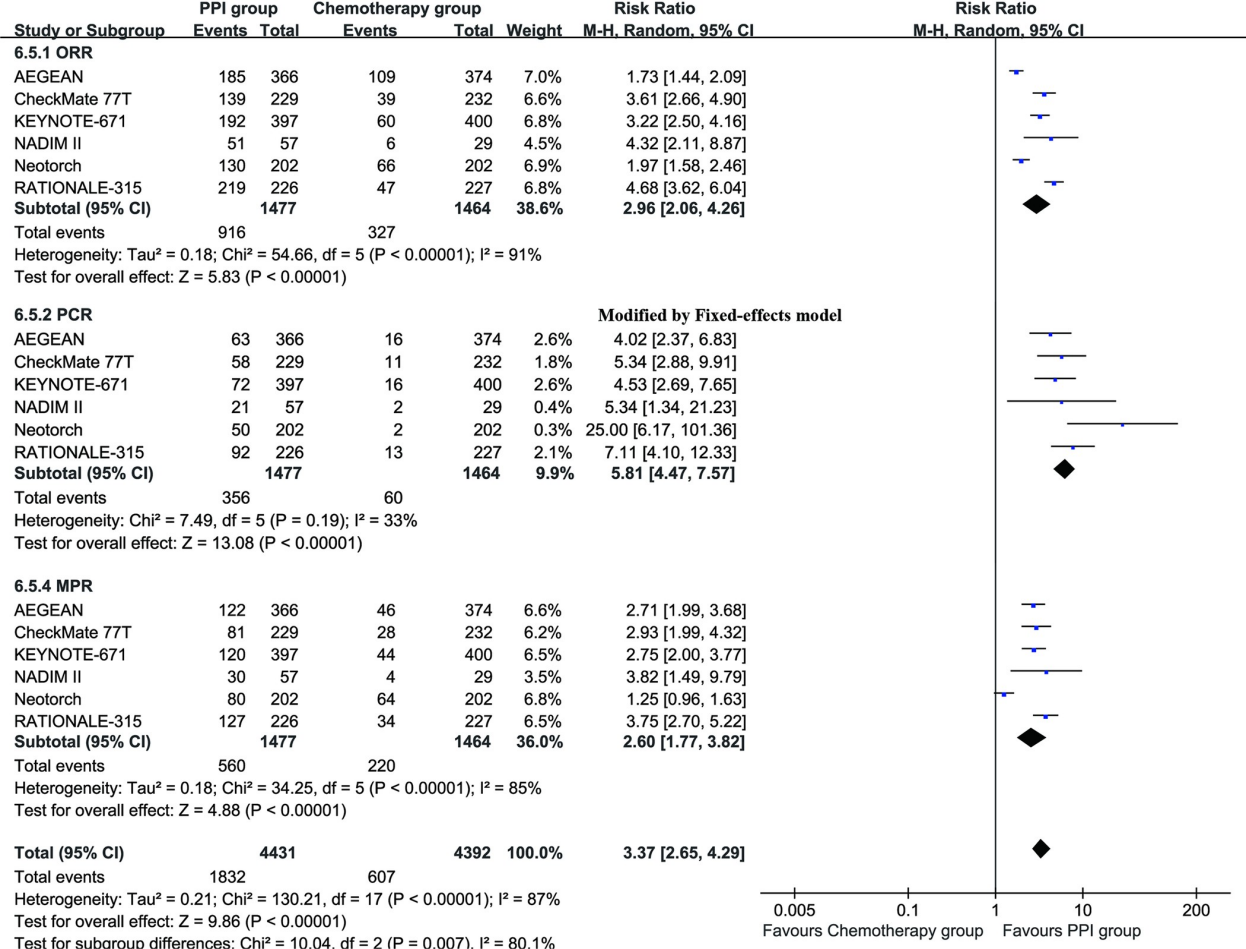
Forest plots of pathological responses associated with PPI versus chemotherapy.

The ORR (RR: 2.96 [2.06, 4.26]), pathological complete response (PCR) (RR: 5.81 [4.47, 7.57]), and MPR (RR: 2.60 [1.77, 3.82]) were greater in the PPI group (**Fig 4**).

### Surgery summary

In the PPI group, there was an increase in the rates of surgery (RR: 1.05 [1.01, 1.09]) and R0 resection (RR: 1.10 [1.05, 1.15]) (**S4 Fig**).

### Safety

Overall, the PPI group had increased rates of grade 3–5 AEs (RR: 1.12 [1.04, 1.20]), serious AEs (RR: 1.34 [1.19, 1.51]), fatal AEs (RR: 1.64 [1.00, 2.68]), and discontinuations due to AEs (RR: 1.93 [1.54, 2.41]). The chemotherapy group showed a tendency for higher total AEs and dose interruptions due to AEs, but this was not statistically significant (**Table 3 and S5 Fig**).

**Table 3:** Summary of adverse events.

Adverse events	PPI		Chemotherapy		Risk ratio [95% CI]	P
	Event/total	%	Event/total	%		
Total adverse events	1467/1477	99.32%	1431/1464	97.75%	1.01 [0.99, 1.02]	0.31
Grade 3–5 adverse events	759/1477	51.39%	682/1464	46.58%	1.12 [1.04, 1.20]	0.003
Serious adverse events	434/1420	30.56%	328/1435	22.86%	1.34 [1.19, 1.51]	< 0.00001
Fatal adverse events	39/1420	2.75%	24/1435	1.67%	1.64 [1.00, 2.68]	0.05
Discontinuation due to adverse events	202/1420	14.23%	106/1435	7.39%	1.93 [1.54, 2.41]	< 0.00001
Dose interruption due to adverse events	145/428	33.88%	102/429	23.78%	1.50 [0.93, 2.42]	0.09

**Abbreviations:** CI: confidence interval; PD-1: Programmed cell death protein 1; PD-L1: Programmed cell death 1 ligand 1; PPI: Perioperative PD-1/PD-L1 inhibitors.

For any grade AEs, the PPI group experienced more instances of increased AST, constipation, fatigue, cough, increased ALT, hypothyroidism, rash, pruritus, pneumonitis, hyperthyroidism, and thyroiditis (**Table 4 and S4 Table**).

**Table 4:** Any grade adverse events (incidence rate > 10% in the PPI group).

Adverse events	PPI		Chemotherapy		Risk ratio [95% CI]	P
	Event/total	%	Event/total	%		
Anemia	565/1477	38.25%	542/1464	37.02%	1.05 [0.96, 1.14]	0.34
Neutrophil count decreased	443/1218	36.37%	419/1233	33.98%	1.07 [0.97, 1.17]	0.18
Nausea	481/1420	33.87%	477/1435	33.24%	1.02 [0.92, 1.13]	0.72
Neutropenia	182/568	32.04%	177/576	30.73%	1.04 [0.88, 1.22]	0.66
AST increased	117/428	27.34%	78/429	18.18%	1.50 [1.17, 1.94]	0.002
White blood cell count decreased	312/1191	26.20%	304/1203	25.27%	1.03 [0.92, 1.17]	0.6
Leukopenia	144/568	25.35%	127/576	22.05%	1.14 [0.95, 1.37]	0.16
Constipation	297/1194	24.87%	245/1208	20.28%	1.23 [1.06, 1.42]	0.007
Alopecia	336/1477	22.75%	344/1464	23.50%	0.96 [0.85, 1.09]	0.52
Fatigue	282/1251	22.54%	225/1237	18.19%	1.20 [1.03, 1.40]	0.02
Arrhythmia	58/259	22.39%	53/231	22.94%	1.07 [0.78, 1.48]	0.66
Decreased appetite	255/1191	21.41%	232/1203	19.29%	1.14 [0.85, 1.52]	0.4
Peripheral sensory neuropathy	49/259	18.92%	39/231	16.88%	1.06 [0.73, 1.54]	0.76
Cough	101/599	16.86%	74/602	12.29%	1.37 [1.05, 1.79]	0.02
ALT increased	207/1248	16.59%	139/1232	11.28%	1.49 [1.23, 1.81]	< 0.0001
Vomiting	149/965	15.44%	126/976	12.91%	1.19 [0.96, 1.49]	0.11
Platelet count decreased	148/989	14.96%	154/1001	15.38%	0.97 [0.79, 1.19]	0.77
Thrombocytopenia	78/568	13.73%	74/576	12.85%	1.06 [0.80, 1.42]	0.68
Asthenia	93/763	12.19%	109/774	14.08%	0.87 [0.67, 1.12]	0.27
Procedural pain	70/599	11.69%	71/602	11.79%	0.99 [0.73, 1.35]	0.96
Incision site pain	111/965	11.50%	99/976	10.14%	1.13 [0.88, 1.46]	0.33
Hypothyroidism	161/1477	10.90%	28/1464	1.91%	5.66 [3.83, 8.36]	< 0.00001
Insomnia	61/568	10.74%	58/576	10.07%	1.07 [0.76, 1.50]	0.7
Diarrhea	133/1251	10.63%	112/1237	9.05%	1.28 [0.85, 1.93]	0.24
Rash	133/1251	10.63%	63/1237	5.09%	2.08 [1.57, 2.77]	< 0.00001
Pneumonia	67/656	10.21%	63/631	9.98%	1.04 [0.76, 1.43]	0.78

**Abbreviations:** ALT: Alanine Aminotransferase; AST: Aspartate Aminotransferase; CI: confidence interval; PD-1: Programmed cell death protein 1; PD-L1: Programmed cell death 1 ligand 1; PPI: Perioperative PD-1/PD-L1 inhibitors.

For grade 3–5 AEs, the PPI group experienced more instances of pneumonitis and rash. The top 5 grade 3–5 AEs in the PPI group were decreased neutrophil count (23.15%), neutropenia (18.31%), leukopenia (6.87%), anemia (6.23%), and decreased white blood cell count (5.79%) (**Table 5 and S5 Table**).

**Table 5:** Grade 3–5 adverse events (incidence rate > 1% in the PPI group).

Adverse events	PPI		Chemotherapy		Risk ratio [95% CI]	P
	Event/total	%	Event/total	%		
Neutrophil count decreased	282/1218	23.15%	270/1233	21.90%	1.05 [0.92, 1.20]	0.44
Neutropenia	104/568	18.31%	98/576	17.01%	1.07 [0.84, 1.36]	0.58
Leukopenia	39/568	6.87%	28/576	0.05	1.27 [0.53, 3.03]	0.59
Anemia	92/1477	6.23%	87/1464	0.06	1.07 [0.80, 1.42]	0.65
White blood cell count decreased	69/1191	5.79%	67/1203	5.57%	1.04 [0.75, 1.43]	0.82
Pneumonia	33/599	5.51%	29/602	4.82%	1.14 [0.71, 1.84]	0.59
Thrombocytopenia	20/568	3.52%	16/576	0.03	1.26 [0.66, 2.41]	0.48
Platelet count decreased	32/989	3.24%	42/1001	4.20%	0.77 [0.49, 1.21]	0.26
Pneumonitis	16/828	1.93%	5/834	0.60%	3.22 [1.19, 8.74]	0.02
Hyperglycemia	6/431	1.39%	1/434	0.23%	6.00 [0.73, 49.39]	0.10
Vomiting	11/965	1.14%	5/976	0.51%	2.12 [0.77, 5.84]	0.15
ALT increased	13/1248	1.04%	5/1232	0.41%	2.62 [0.94, 7.33]	0.07

**Abbreviations:** ALT: Alanine Aminotransferase; AST: Aspartate Aminotransferase; CI: confidence interval; PD-1: Programmed cell death protein 1; PD-L1: Programmed cell death 1 ligand 1; PPI: Perioperative PD-1/PD-L1 inhibitors.

### Sensitivity analysis

Sensitivity analyses were performed for EFS (former smokers), EFSR at 12 months, and MPR. These analyses revealed that the overall reliability of the findings remained intact when any single study was excluded (**S6 Fig**).

### Publication bias

**Fig 5:**
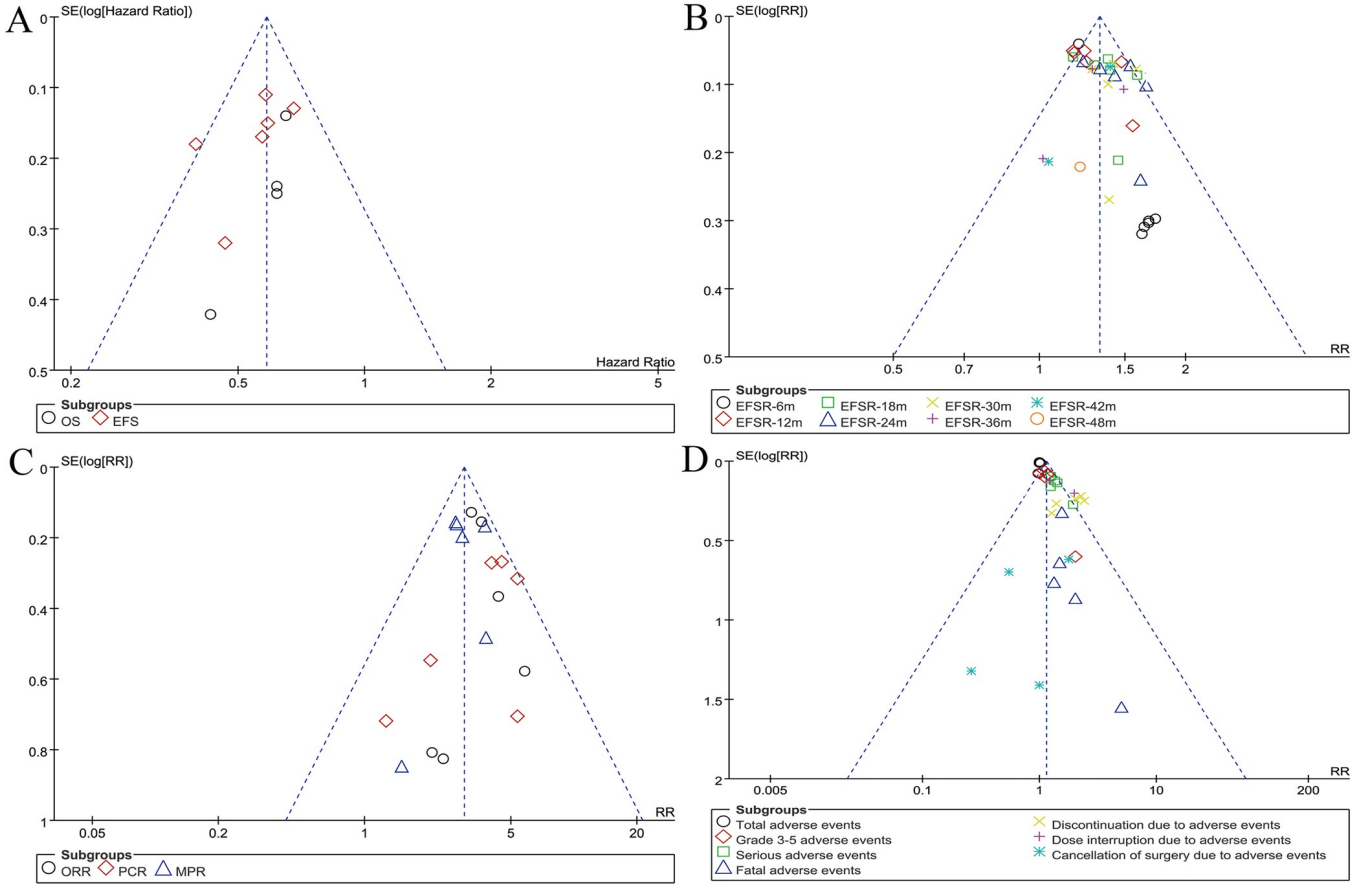
Funnel plots of survival (A), EFSR (B), pathological responses (C), and safety summary (D).

The symmetry observed in funnel plots for survival, EFSR, pathological responses, and the safety summary indicated an acceptable level of publication bias (**Fig 5**).

## Discussion

Various studies have established the efficacy of PI in both neoadjuvant and adjuvant settings [4, 5]. This finding was also corroborated by Pasqualotto et al.’s meta-analysis [6]. However, there is still a lack of large-sample evidence-based medical data for their use in the perioperative setting (neoadjuvant plus adjuvant). Our meta-analysis provides robust evidence supporting the combination of PPI with neoadjuvant chemotherapy for resectable NSCLC. The findings demonstrate significant improvements in OS and EFS, as well as enhanced pathological responses. However, the increased rates of AEs highlight the need for careful patient selection and management strategies to mitigate potential risks.

The pooled data from six RCTs show that the PPI group significantly improves OS and EFS for resectable NSCLC. This benefit was consistent across various subgroups, particularly in patients with a PD-L1 TPS greater than 50%, suggesting that higher PD-L1 expression may predict better responses to PI. These findings are corroborated by recent studies. For example, Forde et al. demonstrated that neoadjuvant nivolumab combined with chemotherapy significantly enhanced PCR and MPR compared to chemotherapy alone, ultimately translating into better survival outcomes [4]. Similarly, Provencio et al. indicated that perioperative PI significantly enhance survival rates in resectable NSCLC [18]. Furthermore, Efil et al. highlighted that the integration of immunotherapy in the perioperative setting leads to a substantial increase in OS and EFS, reinforcing the survival benefit observed in this meta-analysis [19]. The combined effect of chemotherapy and immunotherapy boosts tumor antigen presentation and fosters a stronger anti-tumor immune response [20, 21].

Our analysis also revealed that the PPI regimen significantly increased the rates of PCR and MPR. The RRs for PCR and MPR were 5.81 and 2.60, respectively, indicating that a greater proportion of patients achieved complete or near-complete eradication of their tumors. This is a critical finding, as pathological response has been associated with improved long-term outcomes in NSCLC. Pathological response serves as an important surrogate marker for survival in cancer treatment. Achieving a higher rate of PCR or MPR suggests that the combination therapy is effective in substantially reducing tumor burden, which is likely to translate into lower recurrence rates and better survival outcomes. This is supported by the NADIM trial, which showed that patients achieving PCR with neoadjuvant nivolumab and chemotherapy had significantly better EFS and OS than those who did not achieve PCR [18]. Recent studies have further validated these findings. For instance, the CheckMate 816 trial reported that neoadjuvant nivolumab plus chemotherapy led to a significant increase in MPR and PCR rates, compared to chemotherapy alone, which is consistent with our results [22]. Additionally, Gadgeel et al. highlighted that the enhanced pathological responses observed with the perioperative PPI approach can be attributed to the synergistic effects of chemotherapy and immunotherapy, which enhance tumor antigen presentation and T-cell activation [23].

Despite the efficacy benefits, the higher incidence of grade 3–5 AEs raises concerns about the safety of the PPI regimen. Common severe AEs included pneumonitis and rash, which require vigilant monitoring and management. These findings underscore the need for balancing the potential benefits of PPIs with their associated risks. The safety profile of PI has been well-documented, with immune-related adverse events (irAEs) being a notable concern. These irAEs result from the activation of the immune system against normal tissues, leading to a range of inflammatory conditions that can affect various organs, including the lungs, liver, skin, and endocrine glands [24]. In the context of perioperative treatment, the risk of irAEs must be carefully weighed against the potential survival benefits, especially since these events can greatly affect the patient’s compliance with treatment and overall quality of life [25, 26].

In clinical practice, managing the safety concerns associated with PPIs involves several strategies. Early identification and prompt management of irAEs are crucial to minimizing their severity and preventing long-term complications. This requires regular monitoring of patients, educating them about the potential signs and symptoms of irAEs, and having a clear management plan in place that includes the use of immunosuppressive agents such as corticosteroids when necessary [27]. Furthermore, patient selection is critical to optimizing the safety and efficacy of perioperative PPIs. Identifying biomarkers that can predict response to therapy and the likelihood of developing severe irAEs can help tailor treatment to individual patients, thereby maximizing the therapeutic benefits while minimizing risks. For example, PD-L1 expression levels and other immune-related biomarkers have been investigated as potential predictors of response to PI [28, 29]. The use of these biomarkers can significantly improve the safety profile and efficacy of the treatment regimen [30, 31].

Our study has some limitations. First, restricting the review to English-language publications may have caused language bias. Second, the inclusion of RCTs that were not all phase 3 trials could influence the robustness of the outcomes. Third, the unavailability of individual patient data precluded a detailed meta-analysis, which may have limited the clinical relevance of the findings.

## Conclusion

The PPI group offers significant survival and pathological response benefits for resectable NSCLC. The survival advantages were confirmed across all subgroups and increased with longer survival times. However, the increased risk of severe AEs necessitates careful patient management and further investigation to optimize treatment protocols. Future studies should focus on improving patient selection criteria, developing methods to minimize AEs, and examining the long-term outcomes of this treatment on survival and safety.

## Supporting information

S1 Checklist PRISMA 2020 checklist. (DOCX)

S1 Fig Cochrane risk assessment. (TIF)

S2 Fig Forest plots of OSR at 12–48 months associated with PPI versus chemotherapy. (TIF)

S3 Fig Forest plots of EFSR at 6–48 months associated with PPI versus chemotherapy. (TIF)

S4 Fig Forest plots of surgery summary associated with PPI versus chemotherapy. (TIF)

S5 Fig Forest plots of safety summary associated with PPI versus chemotherapy. (TIF)

S6 Fig Sensitivity analysis of EFS (Smoking status—Former smoker) (A), EFSR-12m (B), and MPR (C).
(TIF)

S1 Table Search strategy. (DOCX)

S2 Table Methodological quality assessments (Jadad scale) of the included studies. (DOC)

S3 Table GRADE quality assessment by therapeutic strategy and study design for the outcomes. (DOC)

S4 Table Any grade adverse events (all). (DOC)

S5 Table Grade 3–5 adverse events (all). (DOC)

S1 File Extract data details. (XLSX)

## Abbreviations

AE: Adverse event
ALT: Alanine aminotransferase
AST: Aspartate aminotransferase
CI: Confidence interval
CR: Complete response
DCR: Disease control rate
ECOG PS: Eastern Cooperative Oncology Group Performance Status
EFS: Event-free survival
EFSR: Event-free survival rate
GRADE: Grading of Recommendations, Assessment, Development, and Evaluation
HR: Hazard ratio
irAEs: Immune-related adverse events
M/F: Male/Female
MPR: Major pathologic response
PCR: Pathological complete response
ORR: Objective response rate
OS: Overall survival
OSR: Overall survival rate
PD-1: Programmed cell death protein 1
PD-L1: Programmed cell death 1 ligand 1
PICOS: Participants, Intervention, Control, Outcomes, Study design
PI: PD-1/PD-L1 inhibitors
PPI: Perioperative PD-1/PD-L1 inhibitors
PR: Partial response
PRISMA: Preferred Reporting Items for Systematic Reviews and Meta-Analyses
RCT: Randomized controlled trial
RR: Risk ratio
SCC: Squamous cell carcinoma
SD: Stable disease
TNM: Tumor Node Metastasis
TPS: Tumor cell proportion score
TRAEs: Treatment-related adverse events
