# Supplementary figures and images for: Perioperative PD-1/PD-L1 inhibitors for resectable non-small cell lung cancer: A meta-analysis based on randomized controlled trials
Source: PLoS One. 2024 Sep 23;19(9):e0310808. doi: 10.1371/journal.pone.0310808 (PMC11419369; doi:10.1371/journal.pone.0310808)

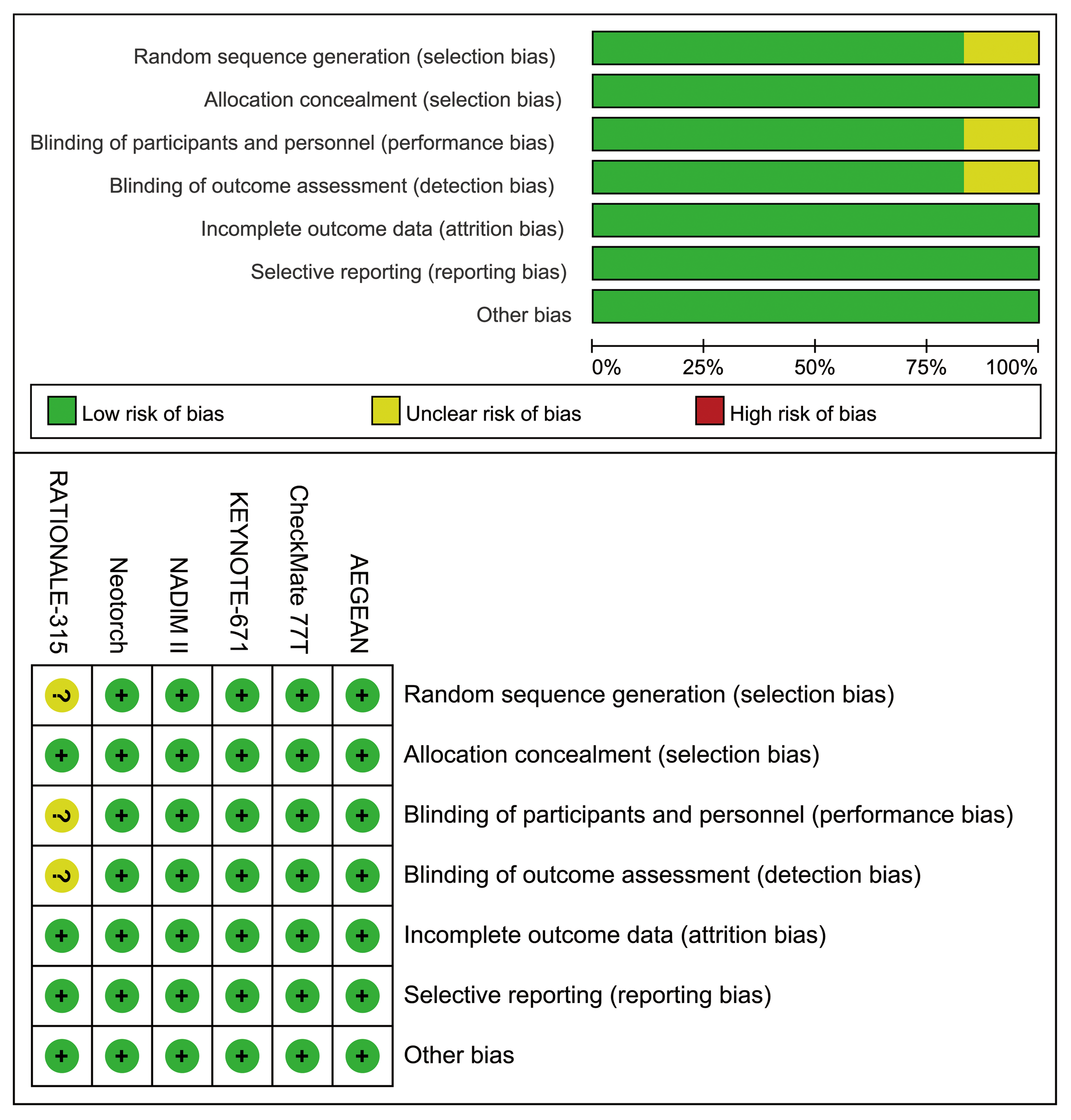

Supplement: S1 Fig — (TIF) [file pone.0310808.s002.tif]

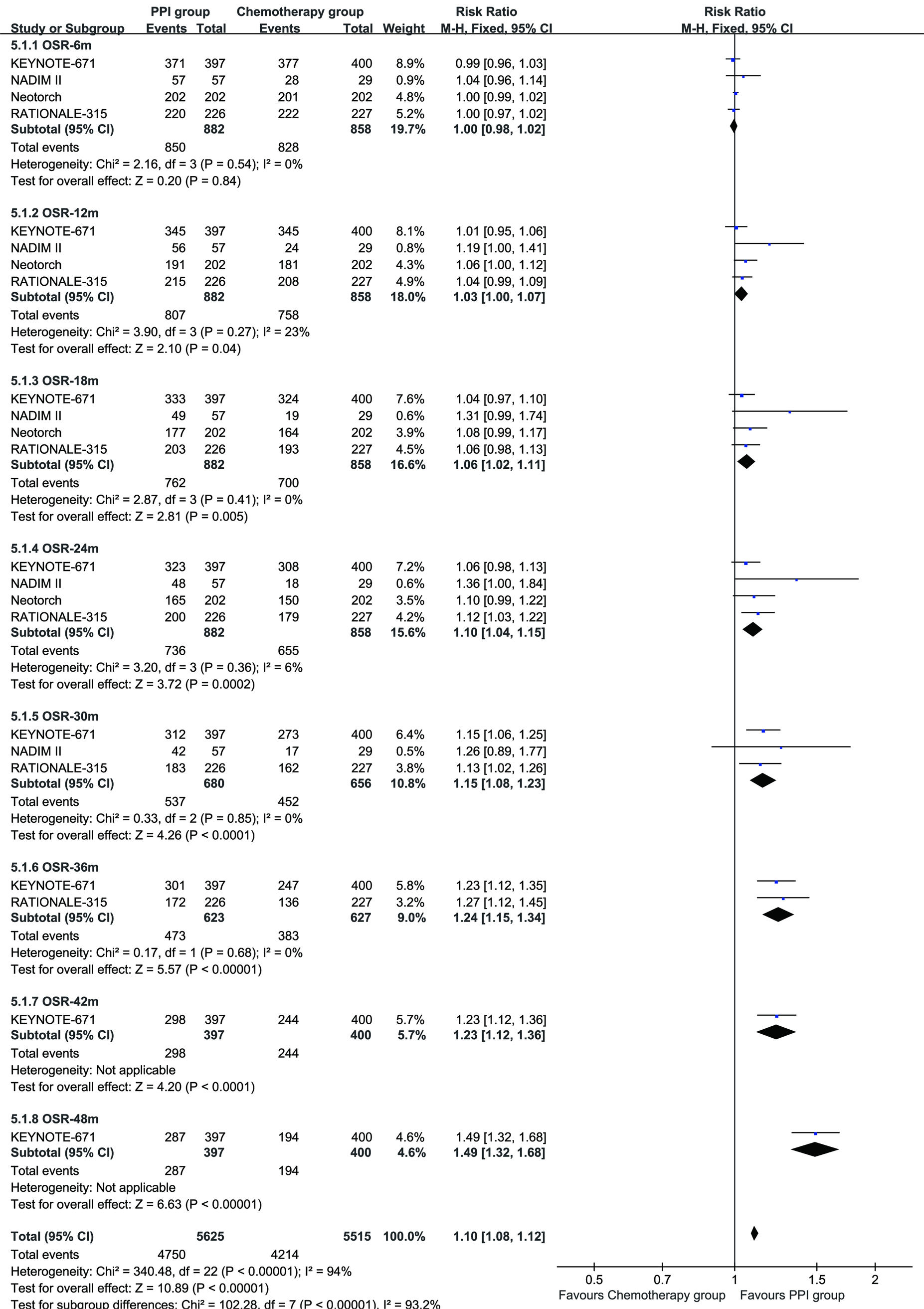

Supplement: S2 Fig — (TIF) [file pone.0310808.s003.tif]

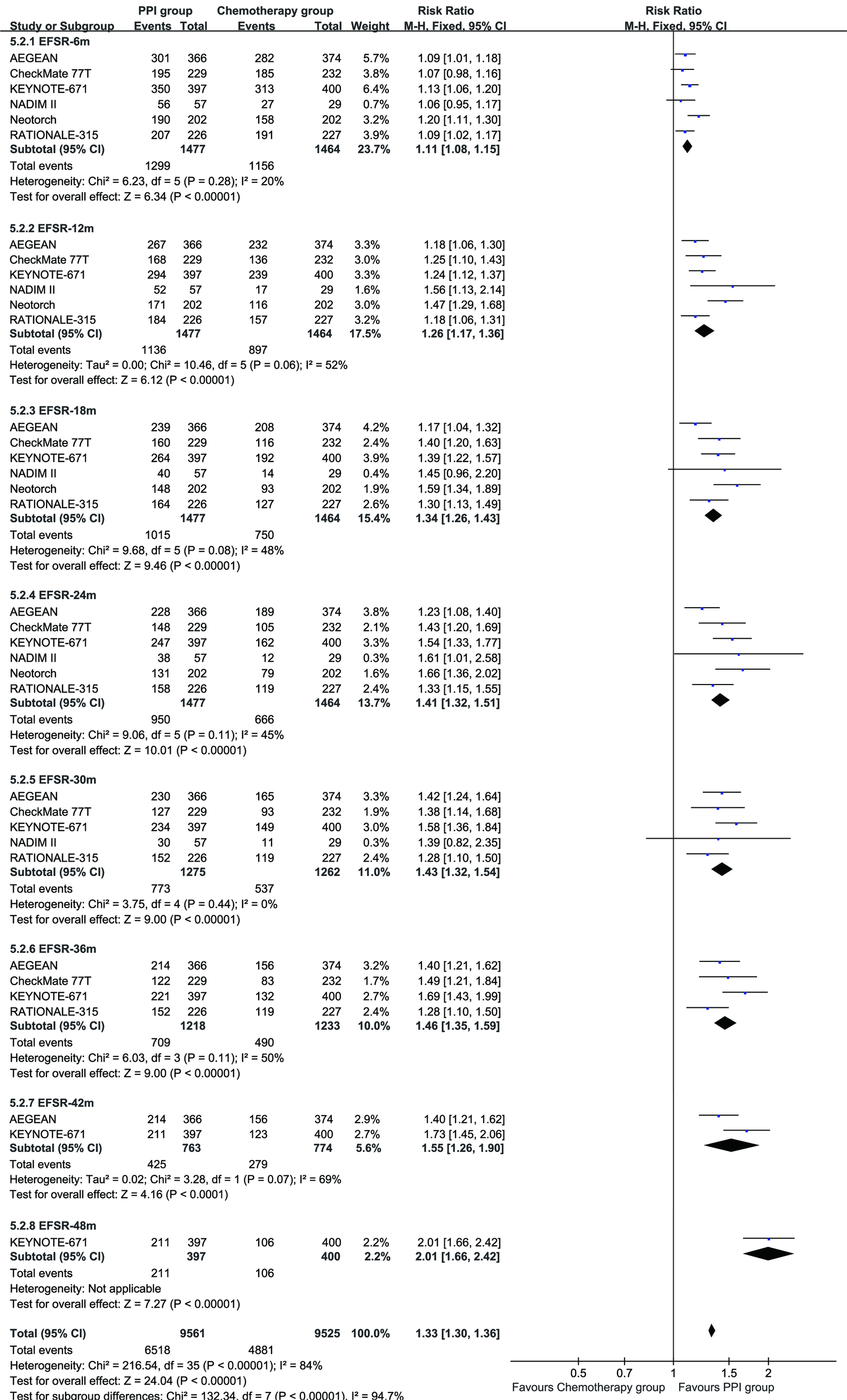

Supplement: S3 Fig — (TIF) [file pone.0310808.s004.tif]

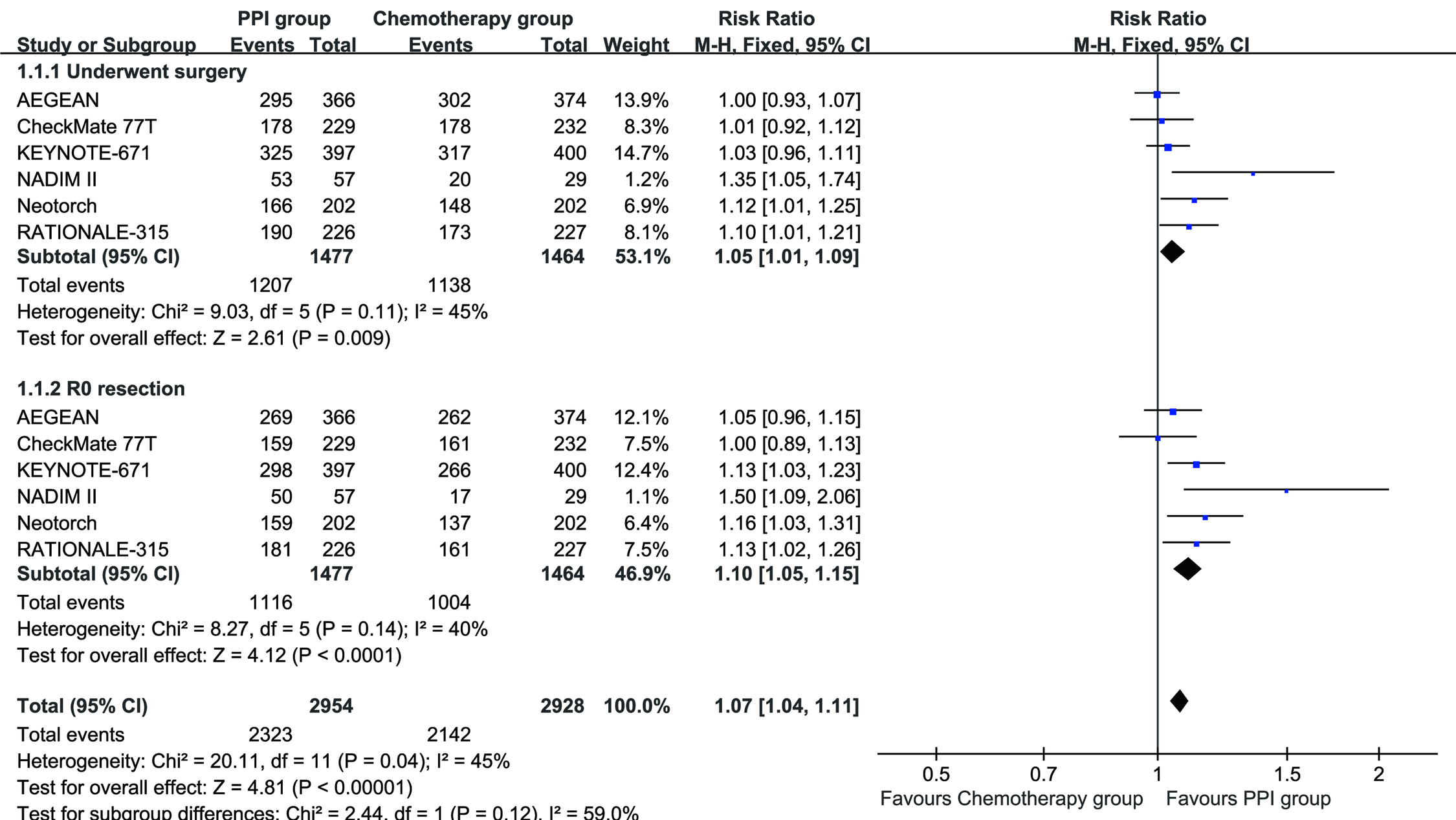

Supplement: S4 Fig — (TIF) [file pone.0310808.s005.tif]

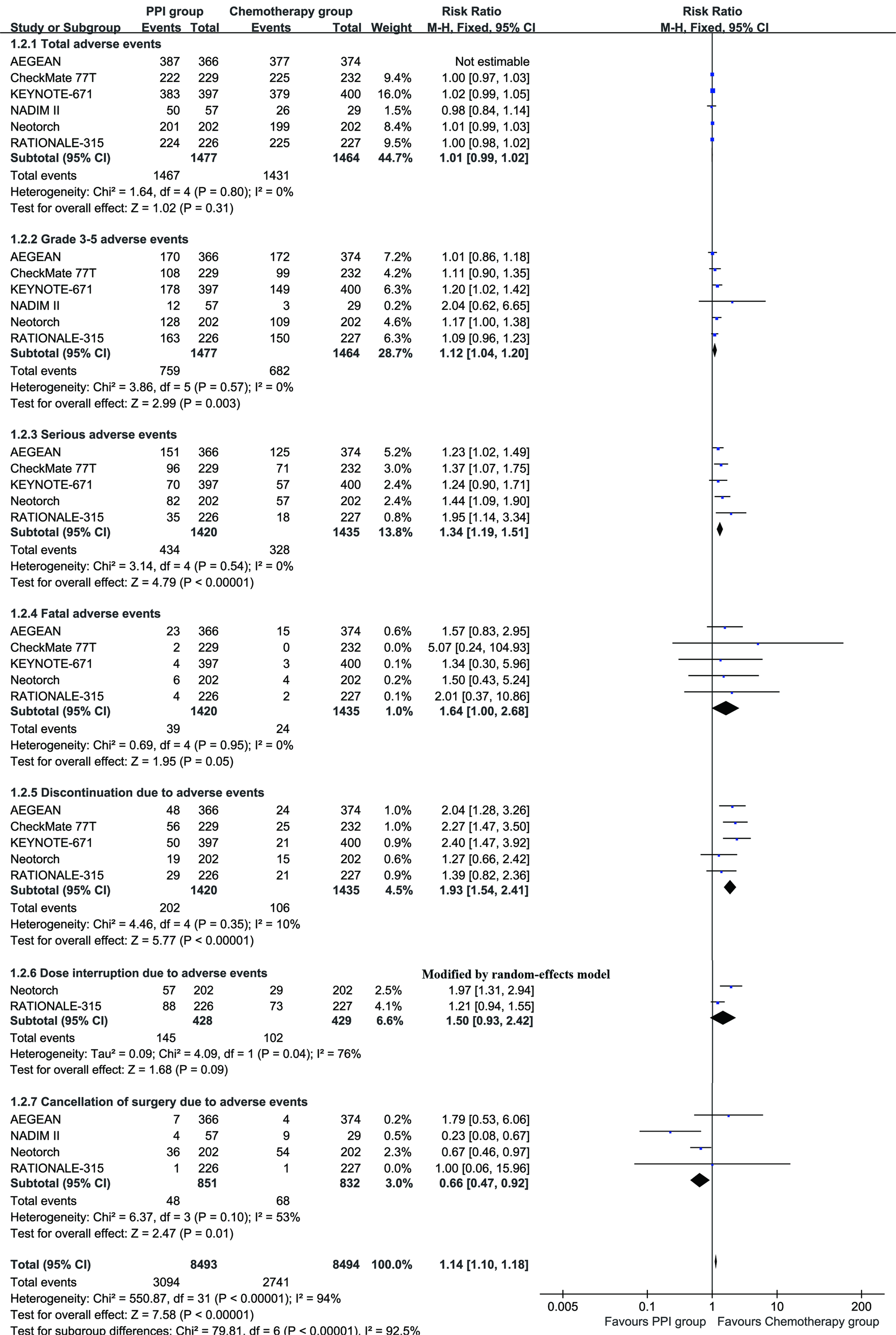

Supplement: S5 Fig — (TIF) [file pone.0310808.s006.tif]

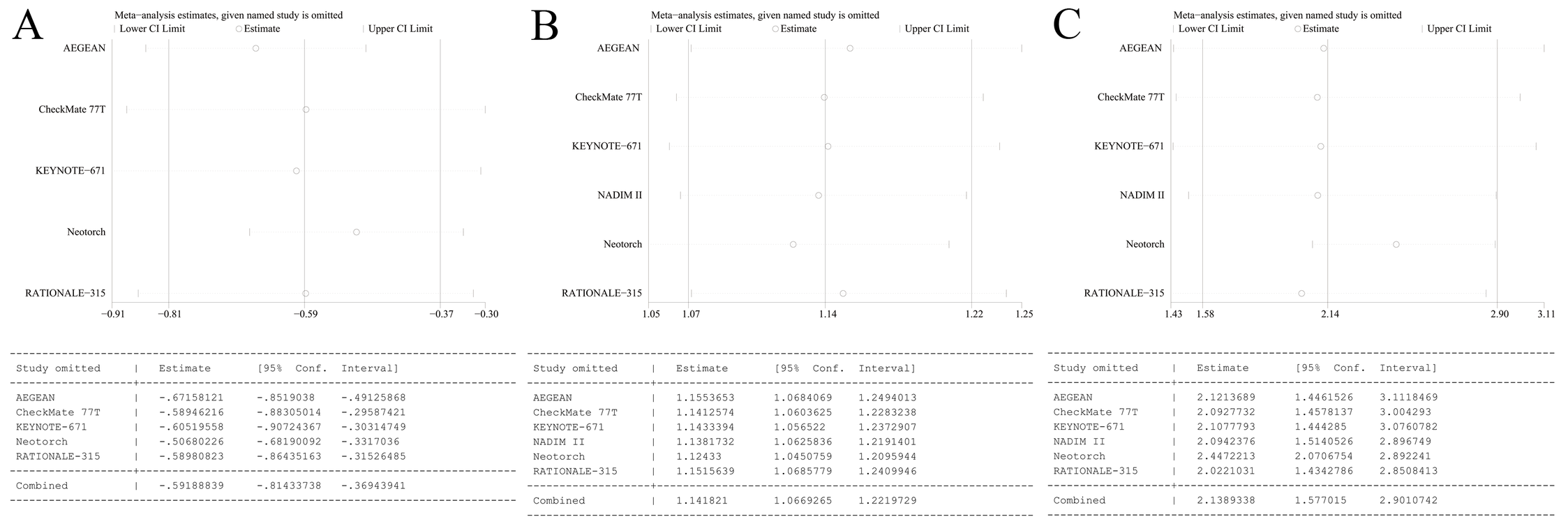

Supplement: S6 Fig — Sensitivity analysis of EFS (Smoking status—Former smoker) (A), EFSR-12m (B), and MPR (C). (TIF) [file pone.0310808.s007.tif]
