# Supplementary material for: Perioperative PD-1/PD-L1 inhibitors for resectable non-small cell lung cancer: A meta-analysis based on randomized controlled trials
Source: PLoS One. 2024 Sep 23;19(9):e0310808. doi: 10.1371/journal.pone.0310808 (PMC11419369; doi:10.1371/journal.pone.0310808)
Supplement: S1 Table — (DOCX) [file pone.0310808.s008.docx]

**S1 Table** Search strategy.

| **PubMed**  The database was searched on June 15, 2024, n=719.  Search Strategy:  **#1 Nivolumab[Title/Abstract] OR Pembrolizumab[Title/Abstract] OR Toripalimab[Title/Abstract] OR Sintilimab[Title/Abstract] OR Camrelizumab[Title/Abstract] OR Tislelizumab[Title/Abstract] OR Penpulimab[Title/Abstract] OR Zimberelimab[Title/Abstract] OR Serplulimab[Title/Abstract] OR Durvalumab[Title/Abstract] OR Atezolizumab[Title/Abstract] OR Envolizumab[Title/Abstract] OR Sugemalimab[Title/Abstract] OR Adebrelimab[Title/Abstract] OR Cemiplimab[Title/Abstract] OR Dostarlimab[Title/Abstract] OR Retifanlimab[Title/Abstract] OR Envafolimab[Title/Abstract] OR Suptavumab[Title/Abstract] OR Cadonilimab[Title/Abstract] OR**  Dostarlimab**[Title/Abstract] OR** Retifanlimab**[Title/Abstract] Sort by: Most Recent n = 21552**  **#2 Chemotherapy [Title/Abstract] Sort by: Most Recent n = 469580**  **#3 Lung cancer[Title/Abstract] OR NSCLC[Title/Abstract] OR Lung adenocarcinoma[Title/Abstract] OR Lung squamous cell carcinoma[Title/Abstract] Sort by: Most Recent n = 234604**  **#4 Randomized[Title/Abstract]) OR Randomly[Title/Abstract] OR Randomised[Title/Abstract] n = 1177825**  **#1 and #2 and #3 and #4 n = 478** |
| --- |
| **Web of Science**  The database was searched on June 15, 2024, 2024, n=486.  Search Strategy:  (Nivolumab(Abstract) OR Pembrolizumab(Abstract) OR Toripalimab(Abstract) OR Sintilimab(Abstract) OR Camrelizumab(Abstract) OR Tislelizumab(Abstract) OR Penpulimab(Abstract) OR Zimberelimab(Abstract) OR Serplulimab(Abstract) OR Durvalumab(Abstract) OR Atezolizumab(Abstract) OR Envolizumab(Abstract) OR Sugemalimab(Abstract) OR Adebrelimab(Abstract) OR Cemiplimab(Abstract) OR Envafolimab(Abstract) OR Suptavumab(Abstract) OR Cadonilimab(Abstract) OR Dostarlimab(Abstract) OR Retifanlimab(Abstract)) AND (**Chemotherapy**(Abstract)) AND **(**Lung cancer(Abstract) **OR** NSCLC(Abstract) **OR**  Lung adenocarcinoma(Abstract**)) OR**  Lung squamous cell carcinoma(Abstract**)**) AND **(Randomly**(Abstract) **OR Randomised**(Abstract**) OR**  **Randomized** (Abstract**))** |
| **EMBASE**  The database was searched on June 15, 2024, 2024, n=1432.  Search Strategy:  (Nivolumab:ti,ab,kw OR Pembrolizumab:ti,ab,kw OR Toripalimab:ti,ab,kw OR Sintilimab:ti,ab,kw OR Camrelizumab:ti,ab,kw OR Tislelizumab:ti,ab,kw OR Penpulimab:ti,ab,kw OR Zimberelimab:ti,ab,kw OR Serplulimab:ti,ab,kw OR Durvalumab:ti,ab,kw OR Atezolizumab:ti,ab,kw OR Envolizumab:ti,ab,kw OR Sugemalimab:ti,ab,kw OR Adebrelimab:ti,ab,kw OR Cemiplimab:ti,ab,kw OR Envafolimab:ti,ab,kw OR Suptavumab:ti,ab,kw OR Cadonilimab:ti,ab,kw OR Dostarlimab:ti,ab,kw OR Retifanlimab:ti,ab,kw) AND (**Chemotherapy**:ti,ab,kw) AND **(**Lung cancer:ti,ab,kw **OR** NSCLC:ti,ab,kw **OR** Lung adenocarcinoma:ti,ab,kw**) OR** Lung squamous cell carcinoma:ti,ab,kw) AND **(Randomly**:ti,ab,kw **OR Randomised**:ti,ab,kw **OR**  **Randomized** :ti,ab,kw**)** |
| **Cochrane Library**  The database was searched on June 15, 2024, 2024, n=85.  Search Strategy:  (Nivolumab OR Pembrolizumab OR Toripalimab OR Sintilimab OR Camrelizumab OR Tislelizumab OR Penpulimab OR Zimberelimab OR Serplulimab OR Durvalumab OR Atezolizumab OR Envolizumab OR Sugemalimab OR Adebrelimab OR Cemiplimab OR Dostarlimab OR Retifanlimab OR Envafolimab OR Suptavumab OR Cadonilimab**)** in Title Abstract Keyword AND (**Chemotherapy)** in Title Abstract Keyword AND (Lung cancer OR NSCLC OR Lung adenocarcinoma OR Lung squamous cell carcinoma**)** in Title Abstract Keyword AND (**Randomized OR Randomly OR Randomised)** in Title Abstract Keyword - (Word variations have been searched) |
| **ScienceDirect**  The database was searched on June 15, 2024, n=2767.  Search Strategy:  Title, abstract, keywords: ((“Nivolumab” OR “Pembrolizumab” OR “Toripalimab” OR “Sintilimab” OR “Camrelizumab” OR “Tislelizumab” OR “Penpulimab” OR “Zimberelimab” OR “Serplulimab” OR “Durvalumab” OR “Atezolizumab” OR “Envolizumab” OR “Sugemalimab” OR “Adebrelimab” OR “Cemiplimab” OR “Dostarlimab” OR “Retifanlimab” OR “Envafolimab” OR “Suptavumab” OR “Cadonilimab”) AND (“**Chemotherapy**”) AND (“Lung cancer” OR “NSCLC” OR “Lung adenocarcinoma” OR “Lung squamous cell carcinoma”) AND (“**Randomized**” **OR Randomly**” **OR** “**Randomised**”)) |
| **Scopus**  The database was searched on June 15, 2024, n=1034.  Search Strategy:  (TITLE-ABS-KEY (Nivolumab OR Pembrolizumab OR Toripalimab OR Sintilimab OR Camrelizumab OR Tislelizumab OR Penpulimab OR Zimberelimab OR Serplulimab OR Durvalumab OR Atezolizumab OR Envolizumab OR Sugemalimab OR Adebrelimab OR Cemiplimab OR Dostarlimab OR Retifanlimab OR Envafolimab OR Suptavumab OR Cadonilimab) AND TITLE-ABS-KEY (**Chemotherapy**) AND TITLE-ABS-KEY (Lung cancer OR NSCLC OR Lung adenocarcinoma OR Lung squamous cell carcinoma) AND TITLE-ABS-KEY (**Randomized OR Randomly OR Randomised**)) |

**Note:** The combined text and medical subject heading (MeSH) terms used were: “**PD-1/PD-L1**”, “**Chemotherapy**”, “Lung cancer”, and “**Randomized**”.
