# Supplementary material for: Perioperative PD-1/PD-L1 inhibitors for resectable non-small cell lung cancer: A meta-analysis based on randomized controlled trials
Source: PLoS One. 2024 Sep 23;19(9):e0310808. doi: 10.1371/journal.pone.0310808 (PMC11419369; doi:10.1371/journal.pone.0310808)
Supplement: S2 Table — (DOC) [file pone.0310808.s009.doc]

**S2 Table Methodological quality assessments (Jadad scale) of the included studies.**

| **Study** | | **Randomization** | **Masking** | **Accountability of all patients** | **Quality (score)** |
| --- | --- | --- | --- | --- | --- |
| AEGEAN (NCT03800134) | Heymach 2023 [8] | ** | ** | * | 5 |
| CheckMate 77T (NCT04025879) | Cascone 2024 [9] | ** | ** | * | 5 |
| KEYNOTE-671 (NCT03425643) | Wakelee 2023 [10] | ** | ** | * | 5 |
| NADIM II (NCT03838159) | Provencio 2023 [11] | ** | ** | * | 5 |
| Neotorch (NCT04158440) | Lu 2024 [12] | ** | ** | * | 5 |
| RATIONALE-315 (NCT04379635) | Zhang 2023 [13] | * | ** | * | 4 |
