# Supplementary material for: Perioperative PD-1/PD-L1 inhibitors for resectable non-small cell lung cancer: A meta-analysis based on randomized controlled trials
Source: PLoS One. 2024 Sep 23;19(9):e0310808. doi: 10.1371/journal.pone.0310808 (PMC11419369; doi:10.1371/journal.pone.0310808)
Supplement: S3 Table — (DOC) [file pone.0310808.s010.doc]

**S3 Table** GRADE quality assessment by therapeutic strategy and study design for the outcomes.

| **Primary outcomes** | **No. of Participants** | | **Differences (95%CI) ^a^** | **Quality Assessment** | | | | | **Quality** |
| --- | --- | --- | --- | --- | --- | --- | --- | --- | --- |
|  | **PPI** | **Chemotherapy** |  | **Risk of Bias^b^** | **Inconsistency** | **Indirectness** | **Imprecision** | **Publication Bias^c^** |  |
| **Survival** |  |  |  |  |  |  |  |  |  |
| OS | 882 | 858 | 0.62 [0.51, 0.77] | Low | No inconsistency | No indirectness | No imprecision | Unlikely | High |
| EFS | 1477 | 1464 | 0.57 [0.51, 0.64] | Low | No inconsistency | No indirectness | No imprecision | Unlikely | High |
| **Survival rate** |  |  |  |  |  |  |  |  |  |
| **OSR** |  |  |  |  |  |  |  |  |  |
| OSR-6m | 850/882 | 828/858 | 1.00 [0.98, 1.02] | Low | No inconsistency | No indirectness | No imprecision | Unlikely | High |
| OSR-12m | 807/882 | 758/858 | 1.03 [1.00, 1.07] | Low | No inconsistency | No indirectness | No imprecision | Unlikely | High |
| OSR-18m | 762/882 | 700/858 | 1.06 [1.02, 1.11] | Low | No inconsistency | No indirectness | No imprecision | Unlikely | High |
| OSR-24m | 736/882 | 655/858 | 1.10 [1.04, 1.15] | Low | No inconsistency | No indirectness | No imprecision | Unlikely | High |
| OSR-30m | 537/680 | 452/656 | 1.15 [1.08, 1.23] | Low | No inconsistency | No indirectness | No imprecision | Unlikely | High |
| OSR-36m | 473/623 | 383/627 | 1.24 [1.15, 1.34] | Low | No inconsistency | No indirectness | No imprecision | Unlikely | High |
| OSR-42m | 298/397 | 244/400 | 1.23 [1.12, 1.36] | Low | No inconsistency | No indirectness | No imprecision | Unlikely | High |
| OSR-48m | 287/397 | 194/400 | 1.49 [1.32, 1.68] | Low | No inconsistency | No indirectness | No imprecision | Unlikely | High |
| **EFSR** |  |  |  |  |  |  |  |  |  |
| EFSR-6m | 1299/1477 | 1156/1464 | 1.11 [1.08, 1.15] | Low | No inconsistency | No indirectness | No imprecision | Unlikely | High |
| EFSR-12m | 1136/1477 | 897/1464 | 1.26 [1.17, 1.36] | Low | Serious (-1) | No indirectness | No imprecision | Unlikely | Medium |
| EFSR-18m | 1015/1477 | 750/1464 | 1.34 [1.26, 1.43] | Low | No inconsistency | No indirectness | No imprecision | Unlikely | High |
| EFSR-24m | 950/1477 | 666/1464 | 1.41 [1.32, 1.51] | Low | No inconsistency | No indirectness | No imprecision | Unlikely | High |
| EFSR-30m | 773/1275 | 537/1262 | 1.43 [1.32, 1.54] | Low | No inconsistency | No indirectness | No imprecision | Unlikely | High |
| EFSR-36m | 709/1218 | 490/1233 | 1.46 [1.35, 1.59] | Low | No inconsistency | No indirectness | No imprecision | Unlikely | High |
| EFSR-42m | 425/763 | 279/774 | 1.55 [1.26, 1.90] | Low | Serious (-1) | No indirectness | No imprecision | Unlikely | Medium |
| EFSR-48m | 211/397 | 106/400 | 2.01 [1.66, 2.42] | Low | No inconsistency | No indirectness | No imprecision | Unlikely | High |
| **Surgery summary** |  |  |  |  |  |  |  |  |  |
| Underwent surgery | 1207/1477 | 1138/1464 | 1.05 [1.01, 1.09] | Low | No inconsistency | No indirectness | No imprecision | Unlikely | High |
| R0 resection | 1116/1477 | 1004/1464 | 1.10 [1.05, 1.15] | Low | No inconsistency | No indirectness | No imprecision | Unlikely | High |
| **Pathological responses** |  |  |  |  |  |  |  |  |  |
| ORR | 916/1477 | 327/1464 | 2.96 [2.06, 4.26] | Low | Serious (-1) | No indirectness | No imprecision | Unlikely | Medium |
| PCR | 356/1477 | 60/1464 | 5.81 [4.47, 7.57] | Low | No inconsistency | No indirectness | No imprecision | Unlikely | High |
| MPR | 560/1477 | 220/1464 | 2.60 [1.77, 3.82] | Low | Serious (-1) | No indirectness | No imprecision | Unlikely | Medium |
| **Safety** |  |  |  |  |  |  |  |  |  |
| **Safety summary** |  |  |  |  |  |  |  |  |  |
| Total adverse events | 1467/1477 | 1431/1464 | 1.01 [0.99, 1.02] | Low | No inconsistency | No indirectness | No imprecision | Unlikely | High |
| Grade 3-5 adverse events | 759/1477 | 682/1464 | 1.12 [1.04, 1.20] | Low | No inconsistency | No indirectness | No imprecision | Unlikely | High |
| Serious adverse events | 434/1420 | 328/1435 | 1.34 [1.19, 1.51] | Low | No inconsistency | No indirectness | No imprecision | Unlikely | High |
| Fatal adverse events | 39/1420 | 24/1435 | 1.64 [1.00, 2.68] | Low | No inconsistency | No indirectness | No imprecision | Unlikely | High |
| Discontinuation due to adverse events | 202/1420 | 106/1435 | 1.93 [1.54, 2.41] | Low | No inconsistency | No indirectness | No imprecision | Unlikely | High |
| Dose interruption due to adverse events | 145/428 | 102/429 | 1.50 [0.93, 2.42] | Low | Serious (-1) | No indirectness | No imprecision | Unlikely | Medium |
| **Any grade adverse Events** |  |  |  |  |  |  |  |  |  |
| Anemia | 565/1477 | 542/1464 | 1.05 [0.96, 1.14] | Low | No inconsistency | No indirectness | No imprecision | Unlikely | High |
| Neutrophil count decreased | 443/1218 | 419/1233 | 1.07 [0.97, 1.17] | Low | No inconsistency | No indirectness | No imprecision | Unlikely | High |
| Nausea | 481/1420 | 477/1435 | 1.02 [0.92, 1.13] | Low | No inconsistency | No indirectness | No imprecision | Unlikely | High |
| Neutropenia | 182/568 | 177/576 | 1.04 [0.88, 1.22] | Low | No inconsistency | No indirectness | No imprecision | Unlikely | High |
| AST increased | 117/428 | 78/429 | 1.50 [1.17, 1.94] | Low | No inconsistency | No indirectness | No imprecision | Unlikely | High |
| White blood cell count decreased | 312/1191 | 304/1203 | 1.03 [0.92, 1.17] | Low | No inconsistency | No indirectness | No imprecision | Unlikely | High |
| Leukopenia | 144/568 | 127/576 | 1.14 [0.95, 1.37] | Low | No inconsistency | No indirectness | No imprecision | Unlikely | High |
| Constipation | 297/1194 | 245/1208 | 1.23 [1.06, 1.42] | Low | No inconsistency | No indirectness | No imprecision | Unlikely | High |
| Alopecia | 336/1477 | 344/1464 | 0.96 [0.85, 1.09] | Low | No inconsistency | No indirectness | No imprecision | Unlikely | High |
| Fatigue | 282/1251 | 225/1237 | 1.20 [1.03, 1.40] | Low | No inconsistency | No indirectness | No imprecision | Unlikely | High |
| Arrhythmia | 58/259 | 53/231 | 1.07 [0.78, 1.48] | Low | No inconsistency | No indirectness | No imprecision | Unlikely | High |
| Decreased appetite | 255/1191 | 232/1203 | 1.14 [0.85, 1.52] | Low | Serious (-1) | No indirectness | No imprecision | Unlikely | Medium |
| Peripheral sensory neuropathy | 49/259 | 39/231 | 1.06 [0.73, 1.54] | Low | No inconsistency | No indirectness | No imprecision | Unlikely | High |
| Cough | 101/599 | 74/602 | 1.37 [1.05, 1.79] | Low | No inconsistency | No indirectness | No imprecision | Unlikely | High |
| ALT increased | 207/1248 | 139/1232 | 1.49 [1.23, 1.81] | Low | No inconsistency | No indirectness | No imprecision | Unlikely | High |
| Vomiting | 149/965 | 126/976 | 1.19 [0.96, 1.49] | Low | No inconsistency | No indirectness | No imprecision | Unlikely | High |
| Platelet count decreased | 148/989 | 154/1001 | 0.97 [0.79, 1.19] | Low | No inconsistency | No indirectness | No imprecision | Unlikely | High |
| Thrombocytopenia | 78/568 | 74/576 | 1.06 [0.80, 1.42] | Low | No inconsistency | No indirectness | No imprecision | Unlikely | High |
| Asthenia | 93/763 | 109/774 | 0.87 [0.67, 1.12] | Low | No inconsistency | No indirectness | No imprecision | Unlikely | High |
| Procedural pain | 70/599 | 71/602 | 0.99 [0.73, 1.35] | Low | No inconsistency | No indirectness | No imprecision | Unlikely | High |
| Incision site pain | 111/965 | 99/976 | 1.13 [0.88, 1.46] | Low | No inconsistency | No indirectness | No imprecision | Unlikely | High |
| Hypothyroidism | 161/1477 | 28/1464 | 5.66 [3.83, 8.36] | Low | No inconsistency | No indirectness | No imprecision | Unlikely | High |
| Insomnia | 61/568 | 58/576 | 1.07 [0.76, 1.50] | Low | No inconsistency | No indirectness | No imprecision | Unlikely | High |
| Diarrhea | 133/1251 | 112/1237 | 1.28 [0.85, 1.93] | Low | Serious (-1) | No indirectness | No imprecision | Unlikely | Medium |
| Rash | 133/1251 | 63/1237 | 2.08 [1.57, 2.77] | Low | No inconsistency | No indirectness | No imprecision | Unlikely | High |
| Pneumonia | 67/656 | 63/631 | 1.04 [0.76, 1.43] | Low | No inconsistency | No indirectness | No imprecision | Unlikely | High |
| Pruritus | 95/1022 | 38/1005 | 2.43 [1.69, 3.50] | Low | No inconsistency | No indirectness | No imprecision | Unlikely | High |
| Dyspnea | 54/599 | 25/602 | 2.17 [1.37, 3.44] | Low | No inconsistency | No indirectness | No imprecision | Unlikely | High |
| Arthralgia | 38/423 | 28/403 | 1.08 [0.68, 1.70] | Low | No inconsistency | No indirectness | No imprecision | Unlikely | High |
| Chest pain | 50/599 | 36/602 | 1.39 [0.93, 2.09] | Low | No inconsistency | No indirectness | No imprecision | Unlikely | High |
| Wound complication | 50/599 | 50/602 | 1.00 [0.69, 1.45] | Low | No inconsistency | No indirectness | No imprecision | Unlikely | High |
| Hyperglycemia | 35/431 | 28/434 | 1.25 [0.79, 1.97] | Low | No inconsistency | No indirectness | No imprecision | Unlikely | High |
| Pneumonitis | 53/828 | 24/834 | 2.22 [1.39, 3.53] | Low | No inconsistency | No indirectness | No imprecision | Unlikely | High |
| Hyperthyroidism | 67/1054 | 26/1061 | 2.59 [1.66, 4.04] | Low | No inconsistency | No indirectness | No imprecision | Unlikely | High |
| Pneumothorax | 28/454 | 27/429 | 0.99 [0.59, 1.65] | Low | No inconsistency | No indirectness | No imprecision | Unlikely | High |
| Pleural effusion | 33/656 | 24/631 | 1.73 [0.52, 5.68] | Low | Serious (-1) | No indirectness | No imprecision | Unlikely | Medium |
| Subcutaneous emphysema | 9/454 | 18/429 | 0.49 [0.22, 1.07] | Low | No inconsistency | No indirectness | No imprecision | Unlikely | High |
| Thyroiditis | 8/623 | 1/627 | 5.70 [1.01, 32.25] | Low | No inconsistency | No indirectness | No imprecision | Unlikely | High |
| Adrenal insufficiency | 8/852 | 0/859 | 6.39 [1.15, 35.63] | Low | No inconsistency | No indirectness | No imprecision | Unlikely | High |
| Hypophysitis | 3/626 | 1/632 | 2.36 [0.35, 15.91] | Low | No inconsistency | No indirectness | No imprecision | Unlikely | High |
| Hepatitis | 2/626 | 3/632 | 0.72 [0.14, 3.64] | Low | No inconsistency | No indirectness | No imprecision | Unlikely | High |
| **Grade 3-5 adverse Events** |  |  |  |  |  |  |  |  |  |
| Neutrophil count decreased | 282/1218 | 270/1233 | 1.05 [0.92, 1.20] | Low | No inconsistency | No indirectness | No imprecision | Unlikely | High |
| Neutropenia | 104/568 | 98/576 | 1.07 [0.84, 1.36] | Low | No inconsistency | No indirectness | No imprecision | Unlikely | High |
| Leukopenia | 39/568 | 28/576 | 1.27 [0.53, 3.03] | Low | Serious (-1) | No indirectness | No imprecision | Unlikely | Medium |
| Anemia | 92/1477 | 87/1464 | 1.07 [0.80, 1.42] | Low | No inconsistency | No indirectness | No imprecision | Unlikely | High |
| White blood cell count decreased | 69/1191 | 67/1203 | 1.04 [0.75, 1.43] | Low | No inconsistency | No indirectness | No imprecision | Unlikely | High |
| Pneumonia | 33/599 | 29/602 | 1.14 [0.71, 1.84] | Low | No inconsistency | No indirectness | No imprecision | Unlikely | High |
| Thrombocytopenia | 20/568 | 16/576 | 1.26 [0.66, 2.41] | Low | No inconsistency | No indirectness | No imprecision | Unlikely | High |
| Platelet count decreased | 32/989 | 42/1001 | 0.77 [0.49, 1.21] | Low | No inconsistency | No indirectness | No imprecision | Unlikely | High |
| Pneumonitis | 16/828 | 5/834 | 3.22 [1.19, 8.74] | Low | No inconsistency | No indirectness | No imprecision | Unlikely | High |
| Hyperglycemia | 6/431 | 1/434 | 6.00 [0.73, 49.39] | Low | No inconsistency | No indirectness | No imprecision | Unlikely | High |
| Vomiting | 11/965 | 5/976 | 2.12 [0.77, 5.84] | Low | No inconsistency | No indirectness | No imprecision | Unlikely | High |
| ALT increased | 13/1248 | 5/1232 | 2.62 [0.94, 7.33] | Low | No inconsistency | No indirectness | No imprecision | Unlikely | High |
| Fatigue | 12/1251 | 9/1237 | 1.22 [0.52, 2.82] | Low | No inconsistency | No indirectness | No imprecision | Unlikely | High |
| Diarrhea | 12/1251 | 6/1237 | 1.68 [0.68, 4.13] | Low | No inconsistency | No indirectness | No imprecision | Unlikely | High |
| AST increased | 4/428 | 0/429 | 5.01 [0.59, 42.71] | Low | No inconsistency | No indirectness | No imprecision | Unlikely | High |
| Nausea | 13/1420 | 10/1435 | 1.28 [0.59, 2.82] | Low | No inconsistency | No indirectness | No imprecision | Unlikely | High |
| Rash | 10/1251 | 1/1237 | 4.65 [1.18, 18.23] | Low | No inconsistency | No indirectness | No imprecision | Unlikely | High |
| Decreased appetite | 8/1191 | 1/1203 | 3.84 [0.95, 15.44] | Low | No inconsistency | No indirectness | No imprecision | Unlikely | High |
| Asthenia | 4/763 | 7/774 | 0.54 [0.02, 12.18] | Low | Serious (-1) | No indirectness | No imprecision | Unlikely | Medium |
| Incision site pain | 5/965 | 3/976 | 1.59 [0.42, 6.06] | Low | No inconsistency | No indirectness | No imprecision | Unlikely | High |
| Dyspnea | 3/599 | 1/602 | 3.02 [0.32, 28.93] | Low | No inconsistency | No indirectness | No imprecision | Unlikely | High |
| Peripheral sensory neuropathy | 1/259 | 2/231 | 0.45 [0.07, 3.01] | Low | No inconsistency | No indirectness | No imprecision | Unlikely | High |
| Pruritus | 3/820 | 0/803 | 4.06 [0.45, 36.23] | Low | No inconsistency | No indirectness | No imprecision | Unlikely | High |
| Constipation | 4/1194 | 1/1208 | 2.23 [0.50, 9.92] | Low | No inconsistency | No indirectness | No imprecision | Unlikely | High |
| Chest pain | 2/599 | 2/602 | 1.00 [0.17, 5.76] | Low | No inconsistency | No indirectness | No imprecision | Unlikely | High |
| Arthralgia | 1/423 | 1/403 | 1.02 [0.06, 16.28] | Low | No inconsistency | No indirectness | No imprecision | Unlikely | High |
| Hyperthyroidism | 2/852 | 0/859 | 3.03 [0.32, 28.98] | Low | No inconsistency | No indirectness | No imprecision | Unlikely | High |
| Adrenal insufficiency | 2/852 | 0/859 | 3.02 [0.31, 28.93] | Low | No inconsistency | No indirectness | No imprecision | Unlikely | High |
| Cough | 1/599 | 1/602 | 1.00 [0.06, 15.88] | Low | No inconsistency | No indirectness | No imprecision | Unlikely | High |
| Hypophysitis | 1/626 | 1/632 | 1.01 [0.14, 7.16] | Low | No inconsistency | No indirectness | No imprecision | Unlikely | High |
| Alopecia | 2/1477 | 3/1464 | 0.79 [0.19, 3.18] | Low | No inconsistency | No indirectness | No imprecision | Unlikely | High |
| Hypothyroidism | 2/1477 | 0/1464 | 5.02 [0.24, 104.02] | Low | No inconsistency | No indirectness | No imprecision | Unlikely | High |
| Hepatitis | 0/626 | 2/632 | 0.20 [0.01, 4.18] | Low | No inconsistency | No indirectness | No imprecision | Unlikely | High |

**Abbreviations:** AE: Adverse event; ALT: Alanine aminotransferase; AST: Aspartate aminotransferase; CI: Confidence interval; CR: Complete response; ECOG PS: Eastern Cooperative Oncology Group Performance Status; EFS: Event-free survival; EFSR: Event-free survival rate; GRADE: Grading of Recommendations, Assessment, Development, and Evaluation; HR: Hazard ratio; MPR: Major pathologic response; PCR: Pathological complete response; ORR: Objective response rate; OS: Overall survival; OSR: Overall survival rate; PD-1: Programmed cell death protein 1; PD-L1: Programmed cell death 1 ligand 1; PPI: Perioperative PD-1/PD-L1 inhibitors; RCT: Randomized controlled trial; RR: Risk ratio; TPS: Tumor cell proportion score; TRAEs: Treatment-related adverse events.

^a^ Differences: hazard ratio (HR) for OS and EFS; risk ratios (RR) for OSR, EFSR, adverse events.

^b^ Risk of bias assessed using the Jadad scale for randomized controlled trials.

^c^ Publication bias was explored through visual inspection of the funnel plots.
