# Supplementary material for: Perioperative PD-1/PD-L1 inhibitors for resectable non-small cell lung cancer: A meta-analysis based on randomized controlled trials
Source: PLoS One. 2024 Sep 23;19(9):e0310808. doi: 10.1371/journal.pone.0310808 (PMC11419369; doi:10.1371/journal.pone.0310808)
Supplement: S4 Table — (DOC) [file pone.0310808.s011.doc]

**S4 Table** Any grade adverse events (all).

| **Adverse events** | **PPI** | |  | **Chemotherapy** | | **Risk ratio [95% CI]** | **P** |
| --- | --- | --- | --- | --- | --- | --- | --- |
|  | **Event/total** | **%** |  | **Event/total** | **%** |  |  |
| Anemia | 565/1477 | 38.25% |  | 542/1464 | 37.02% | 1.05 [0.96, 1.14] | 0.34 |
| Neutrophil count decreased | 443/1218 | 36.37% |  | 419/1233 | 33.98% | 1.07 [0.97, 1.17] | 0.18 |
| Nausea | 481/1420 | 33.87% |  | 477/1435 | 33.24% | 1.02 [0.92, 1.13] | 0.72 |
| Neutropenia | 182/568 | 32.04% |  | 177/576 | 30.73% | 1.04 [0.88, 1.22] | 0.66 |
| AST increased | 117/428 | 27.34% |  | 78/429 | 18.18% | 1.50 [1.17, 1.94] | 0.002 |
| White blood cell count decreased | 312/1191 | 26.20% |  | 304/1203 | 25.27% | 1.03 [0.92, 1.17] | 0.6 |
| Leukopenia | 144/568 | 25.35% |  | 127/576 | 22.05% | 1.14 [0.95, 1.37] | 0.16 |
| Constipation | 297/1194 | 24.87% |  | 245/1208 | 20.28% | 1.23 [1.06, 1.42] | 0.007 |
| Alopecia | 336/1477 | 22.75% |  | 344/1464 | 23.50% | 0.96 [0.85, 1.09] | 0.52 |
| Fatigue | 282/1251 | 22.54% |  | 225/1237 | 18.19% | 1.20 [1.03, 1.40] | 0.02 |
| Arrhythmia | 58/259 | 22.39% |  | 53/231 | 22.94% | 1.07 [0.78, 1.48] | 0.66 |
| Decreased appetite | 255/1191 | 21.41% |  | 232/1203 | 19.29% | 1.14 [0.85, 1.52] | 0.4 |
| Peripheral sensory neuropathy | 49/259 | 18.92% |  | 39/231 | 16.88% | 1.06 [0.73, 1.54] | 0.76 |
| Cough | 101/599 | 16.86% |  | 74/602 | 12.29% | 1.37 [1.05, 1.79] | 0.02 |
| ALT increased | 207/1248 | 16.59% |  | 139/1232 | 11.28% | 1.49 [1.23, 1.81] | < 0.0001 |
| Vomiting | 149/965 | 15.44% |  | 126/976 | 12.91% | 1.19 [0.96, 1.49] | 0.11 |
| Platelet count decreased | 148/989 | 14.96% |  | 154/1001 | 15.38% | 0.97 [0.79, 1.19] | 0.77 |
| Thrombocytopenia | 78/568 | 13.73% |  | 74/576 | 12.85% | 1.06 [0.80, 1.42] | 0.68 |
| Asthenia | 93/763 | 12.19% |  | 109/774 | 14.08% | 0.87 [0.67, 1.12] | 0.27 |
| Procedural pain | 70/599 | 11.69% |  | 71/602 | 11.79% | 0.99 [0.73, 1.35] | 0.96 |
| Incision site pain | 111/965 | 11.50% |  | 99/976 | 10.14% | 1.13 [0.88, 1.46] | 0.33 |
| Hypothyroidism | 161/1477 | 10.90% |  | 28/1464 | 1.91% | 5.66 [3.83, 8.36] | < 0.00001 |
| Insomnia | 61/568 | 10.74% |  | 58/576 | 10.07% | 1.07 [0.76, 1.50] | 0.7 |
| Diarrhea | 133/1251 | 10.63% |  | 112/1237 | 9.05% | 1.28 [0.85, 1.93] | 0.24 |
| Rash | 133/1251 | 10.63% |  | 63/1237 | 5.09% | 2.08 [1.57, 2.77] | < 0.00001 |
| Pneumonia | 67/656 | 10.21% |  | 63/631 | 9.98% | 1.04 [0.76, 1.43] | 0.78 |
| Pruritus | 95/1022 | 9.30% |  | 38/1005 | 3.78% | 2.43 [1.69, 3.50] | < 0.00001 |
| Dyspnea | 54/599 | 9.02% |  | 25/602 | 4.15% | 2.17 [1.37, 3.44] | 0.001 |
| Arthralgia | 38/423 | 8.98% |  | 28/403 | 6.95% | 1.08 [0.68, 1.70] | 0.76 |
| Chest pain | 50/599 | 8.35% |  | 36/602 | 5.98% | 1.39 [0.93, 2.09] | 0.11 |
| Wound complication | 50/599 | 8.35% |  | 50/602 | 8.31% | 1.00 [0.69, 1.45] | 0.99 |
| Hyperglycemia | 35/431 | 8.12% |  | 28/434 | 6.45% | 1.25 [0.79, 1.97] | 0.34 |
| Pneumonitis | 53/828 | 6.40% |  | 24/834 | 2.88% | 2.22 [1.39, 3.53] | 0.0008 |
| Hyperthyroidism | 67/1054 | 6.36% |  | 26/1061 | 2.45% | 2.59 [1.66, 4.04] | < 0.0001 |
| Pneumothorax | 28/454 | 6.17% |  | 27/429 | 6.29% | 0.99 [0.59, 1.65] | 0.96 |
| Pleural effusion | 33/656 | 5.03% |  | 24/631 | 3.80% | 1.73 [0.52, 5.68] | 0.37 |
| Subcutaneous emphysema | 9/454 | 1.98% |  | 18/429 | 4.20% | 0.49 [0.22, 1.07] | 0.07 |
| Thyroiditis | 8/623 | 1.28% |  | 1/627 | 0.16% | 5.70 [1.01, 32.25] | 0.05 |
| Adrenal insufficiency | 8/852 | 0.94% |  | 0/859 | 0.00% | 6.39 [1.15, 35.63] | 0.03 |
| Hypophysitis | 3/626 | 0.48% |  | 1/632 | 0.16% | 2.36 [0.35, 15.91] | 0.38 |
| Hepatitis | 2/626 | 0.32% |  | 3/632 | 0.47% | 0.72 [0.14, 3.64] | 0.69 |

**Abbreviations:** ALT: Alanine Aminotransferase; AST: Aspartate Aminotransferase; CI: confidence interval; PD-1: Programmed cell death protein 1; PD-L1: Programmed cell death 1 ligand 1; PPI: Perioperative PD-1/PD-L1 inhibitors.
