# Supplementary material for: Perioperative PD-1/PD-L1 inhibitors for resectable non-small cell lung cancer: A meta-analysis based on randomized controlled trials
Source: PLoS One. 2024 Sep 23;19(9):e0310808. doi: 10.1371/journal.pone.0310808 (PMC11419369; doi:10.1371/journal.pone.0310808)
Supplement: S5 Table — (DOC) [file pone.0310808.s012.doc]

**S5 Table** Grade 3-5 adverse events (all).

| **Adverse events** | **PPI** | |  | **Chemotherapy** | | **Risk ratio [95% CI]** | **P** |
| --- | --- | --- | --- | --- | --- | --- | --- |
|  | **Event/total** | **%** |  | **Event/total** | **%** |  |  |
| Neutrophil count decreased | 282/1218 | 23.15% |  | 270/1233 | 21.90% | 1.05 [0.92, 1.20] | 0.44 |
| Neutropenia | 104/568 | 18.31% |  | 98/576 | 17.01% | 1.07 [0.84, 1.36] | 0.58 |
| Leukopenia | 39/568 | 6.87% |  | 28/576 | 0.05 | 1.27 [0.53, 3.03] | 0.59 |
| Anemia | 92/1477 | 6.23% |  | 87/1464 | 0.06 | 1.07 [0.80, 1.42] | 0.65 |
| White blood cell count decreased | 69/1191 | 5.79% |  | 67/1203 | 5.57% | 1.04 [0.75, 1.43] | 0.82 |
| Pneumonia | 33/599 | 5.51% |  | 29/602 | 4.82% | 1.14 [0.71, 1.84] | 0.59 |
| Thrombocytopenia | 20/568 | 3.52% |  | 16/576 | 0.03 | 1.26 [0.66, 2.41] | 0.48 |
| Platelet count decreased | 32/989 | 3.24% |  | 42/1001 | 4.20% | 0.77 [0.49, 1.21] | 0.26 |
| Pneumonitis | 16/828 | 1.93% |  | 5/834 | 0.60% | 3.22 [1.19, 8.74] | 0.02 |
| Hyperglycemia | 6/431 | 1.39% |  | 1/434 | 0.23% | 6.00 [0.73, 49.39] | 0.10 |
| Vomiting | 11/965 | 1.14% |  | 5/976 | 0.51% | 2.12 [0.77, 5.84] | 0.15 |
| ALT increased | 13/1248 | 1.04% |  | 5/1232 | 0.41% | 2.62 [0.94, 7.33] | 0.07 |
| Fatigue | 12/1251 | 0.96% |  | 9/1237 | 0.01 | 1.22 [0.52, 2.82] | 0.65 |
| Diarrhea | 12/1251 | 0.96% |  | 6/1237 | 0.49% | 1.68 [0.68, 4.13] | 0.26 |
| AST increased | 4/428 | 0.93% |  | 0/429 | 0.00% | 5.01 [0.59, 42.71] | 0.14 |
| Nausea | 13/1420 | 0.92% |  | 10/1435 | 0.70% | 1.28 [0.59, 2.82] | 0.53 |
| Rash | 10/1251 | 0.80% |  | 1/1237 | 0.08% | 4.65 [1.18, 18.23] | 0.03 |
| Decreased appetite | 8/1191 | 0.67% |  | 1/1203 | 0.08% | 3.84 [0.95, 15.44] | 0.06 |
| Asthenia | 4/763 | 0.52% |  | 7/774 | 0.01 | 0.54 [0.02, 12.18] | 0.69 |
| Incision site pain | 5/965 | 0.52% |  | 3/976 | 0.31% | 1.59 [0.42, 6.06] | 0.50 |
| Dyspnea | 3/599 | 0.50% |  | 1/602 | 0.17% | 3.02 [0.32, 28.93] | 0.34 |
| Peripheral sensory neuropathy | 1/259 | 0.39% |  | 2/231 | 0.01 | 0.45 [0.07, 3.01] | 0.41 |
| Pruritus | 3/820 | 0.37% |  | 0/803 | 0.00 | 4.06 [0.45, 36.23] | 0.21 |
| Constipation | 4/1194 | 0.34% |  | 1/1208 | 0.08% | 2.23 [0.50, 9.92] | 0.29 |
| Chest pain | 2/599 | 0.33% |  | 2/602 | 0.33% | 1.00 [0.17, 5.76] | 1.00 |
| Arthralgia | 1/423 | 0.24% |  | 1/403 | 0.25% | 1.02 [0.06, 16.28] | 0.99 |
| Hyperthyroidism | 2/852 | 0.23% |  | 0/859 | 0.00% | 3.03 [0.32, 28.98] | 0.34 |
| Adrenal insufficiency | 2/852 | 0.23% |  | 0/859 | 0.00% | 3.02 [0.31, 28.93] | 0.34 |
| Cough | 1/599 | 0.17% |  | 1/602 | 0.17% | 1.00 [0.06, 15.88] | 1.00 |
| Hypophysitis | 1/626 | 0.16% |  | 1/632 | 0.16% | 1.01 [0.14, 7.16] | 0.99 |
| Alopecia | 2/1477 | 0.14% |  | 3/1464 | 0.20% | 0.79 [0.19, 3.18] | 0.74 |
| Hypothyroidism | 2/1477 | 0.14% |  | 0/1464 | 0.00% | 5.02 [0.24, 104.02] | 0.30 |
| Hepatitis | 0/626 | 0.00% |  | 2/632 | 0.32% | 0.20 [0.01, 4.18] | 0.30 |

**Abbreviations:** ALT: Alanine Aminotransferase; AST: Aspartate Aminotransferase; CI: confidence interval; PD-1: Programmed cell death protein 1; PD-L1: Programmed cell death 1 ligand 1; PPI: Perioperative PD-1/PD-L1 inhibitors.
